# Chemical Characterization, α-Glucosidase, α-Amylase and Lipase Inhibitory Properties of the Australian Honey Bee Propolis

**DOI:** 10.3390/foods11131964

**Published:** 2022-07-01

**Authors:** Sabah Uddin, Peter R. Brooks, Trong D. Tran

**Affiliations:** 1 School of Science, Technology and Engineering, University of the Sunshine Coast, Maroochydore DC, QLD 4558, Australia; s_u021@student.usc.edu.au (S.U.); pbrooks@usc.edu.au (P.R.B.); 2 Centre for Bioinnovation, University of the Sunshine Coast, Maroochydore DC, QLD 4558, Australia

**Keywords:** Australian honey bee propolis, propolins, antidiabetics, anti-obesity, α-glucosidase, α-amylase, lipase

## Abstract

The use of functional foods and nutraceuticals as a complementary therapy for the prevention and management of type 2 diabetes and obesity has steadily increased over the past few decades. With the aim of exploring the therapeutic potentials of Australian propolis, this study reports the chemical and biological investigation of a propolis sample collected in the Queensland state of Australia which exhibited a potent activity in an in vitro α-glucosidase inhibitory screening. The chemical investigation of the propolis resulted in the identification of six known prenylated flavonoids including propolins C, D, F, G, H, and solophenol D. These compounds potently inhibited the α-glucosidase and two other enzymes associated with diabetes and obesity, α-amylase, and lipase on in vitro and in silico assays. These findings suggest that this propolis is a potential source for the development of a functional food to prevent type 2 diabetes and obesity. The chemical analysis revealed that this propolis possessed a chemical fingerprint relatively similar to the Pacific propolis found in Okinawa (South of Japan), Taiwan, and the Solomon Islands. This is the first time the Pacific propolis has been identified in Australia.

## 1. Introduction

Diabetes mellitus is a chronic metabolic disorder of carbohydrate metabolism characterized by high blood glucose levels [1]. Diabetes is caused by an absolute deficiency in insulin secretion (type 1) or a combination of insulin resistance and insulin secretory defect (type 2) [2], and affects millions of people around the world [3]. In 2017, the International Diabetes Federation Atlas estimated that 425 million people suffered from type 2 diabetes, accounting for approximately 90% of all of the diabetic cases [3]. Around 1.8 million Australians have diabetes, of which 85% are type 2 diabetes [4]. This disease is the biggest challenge threatening Australia’s health system, with an estimated total annual cost impact of approximately $14.6 billion [5].

Studies have acknowledged that two major saccharide hydrolyzing enzymes, α-glucosidase and α-amylase, have an effect on the breakdown and digestion of carbohydrates [6]. The α-glucosidase enzyme is found on the border of the small intestine and acts upon the hydrolysis of α-(1,4) bonds between the monosaccharide units [7]. The α-amylase is present in the saliva in the human body and in some other mammals, and catalyzes the biochemical pathway for the hydrolysis of starch into simple sugars [8]. The inhibition of these two enzymes is considered as an effective treatment for type 2 diabetes, as this would in turn delay or inhibit the breakdown of carbohydrates and the subsequent formation and absorption of glucose after meals. Acarbose, miglitol and voglibose are the current antihyperglycemic drugs used to treat type 2 diabetes [9]. While acarbose inhibits both α-glucosidase and α-amylase, miglitol and voglibose inhibit only α-glucosidase [10]. Although these drugs are effective in the control of postprandial hyperglycemia, they are not suitable for long-term treatment, due to their gastrointestinal side effects [11].

The other risk factor for the development of type 2 diabetes is obesity, resulting in an excessive postprandial hyperglycemia [12,13]. The relationship between obesity and diabetes is based on a progressive defect or decrease in insulin secretion, as well as an increased insulin resistance [14]. Both insulin deficiency and insulin resistance appear prematurely in obese patients, and both worsen similarly towards diabetes [15]. Due to the increasing global prevalence of obesity, the number of patients suffering from various metabolic diseases, including adiposity and type 2 diabetes mellitus, is increasing dramatically [16]. To reduce the prevalence of various metabolic disorders, weight management and obesity prevention are considered as the major objectives by many health organizations. The inhibition of the pancreatic lipase enzyme, which plays a vital role in food fat digestion and absorption, is a means of controlling obesity [17]. A lipase inhibitor, orlistat, is one of the current effective drugs to treat obesity and obesity-associated diseases [18]. However, the side effects related to fat malabsorption, oily fecal spotting, abdominal pain, flatus with discharge, and oily stool diminish its use in the second year of treatment [18].

Over the past two decades, functional foods and natural nutraceuticals have been investigated for the control of various aspects of diabetes mellitus, obesity, and their complications [19,20,21,22,23,24]. Numerous studies have demonstrated the potential health benefits of natural polyphenol compounds against diabetes and obesity, due to their biological properties, including antioxidant activity, increasing thermogenesis, and energy expenditure [25,26]. Propolis (bee glue), which is a pleasantly smelling resinous material produced by honey bees, *Apis mellifera* [27], has been known as a good source of polyphenols, with almost 30 polyphenolic sub-classes identified [28]. Since the main function of propolis is to support the sterility and survivability of the beehive, the protective properties of the bioactive compounds in propolis may have significant benefits for human health [29]. Recent reports have shown clear evidence that some honey bee propolis from around the world possesses unique bioactive compounds suitable for the treatment of diabetes and obesity [16,30]. Due to being considered as a beekeeping waste product, only a few studies on Australian propolis were conducted in the past, which leaves a gap in the knowledge of its therapeutic properties. As part of our efforts to discover the therapeutic values for the Australian honey bee propolis, a subset of our in-house propolis extract library was screened for an α-glucosidase inhibitory activity. As a result, an extract of propolis collected in Queensland, Australia, exhibiting potent inhibition at a concentration of 100 μg/mL was selected for further chemical and biological investigations.

## 2. Materials and Methods

### 2.1. Reagents

All of the reagents including α-glucosidase, dimethyl sulfoxide (DMSO), potassium hydroxide (KOH), phosphate buffer, p-nitrophenyl-glucopyranoside (p-NPG), acarbose, phosphoric acid (H_3_PO_4_), human salivary α-amylase, 2-chloro-4-nitrophenyl-maltotrioside (CNP-G3), tris-HCl, porcine pancreas lipase, p-nitrophenyl butyrate (p-NPB), methanol, orlistat, acetonitrile, deuterated methanol (MeOH-*d*_4_), deuterated chloroform (CHCl_3_-*d*), eriodictyol, naringenin, and quercetin were purchased from Sigma Aldrich. The solvents used for extraction (ethanol—EtOH), HPLC (acetonitrile—MeCN), and LC–MS (MeCN and water—H_2_O) analyses were purchased from Merck. The ultra-pure water used for the HPLC analysis was from an in-house Milli-Q system.

### 2.2. Sample Collection

The study used propolis produced by the European honey bee (*Apis mellifera*). A raw propolis sample was collected from Southeast Queensland, Australia, in 2019 and stored in darkness at 4 °C.

### 2.3. Extraction

The frozen raw propolis sample was powdered by grinding. Fine propolis powder (0.5 g × 2) was kept in 5 mL of 70% (*v*/*v*) EtOH, heated at 65 °C for 30 min and then extracted in an ultrasonic bath for 5 min. The sample was placed in ice for 10 min before being centrifuged at 3600 rpm at 4 °C for 10 min. The supernatant was dried down under vacuum, using a GeneVac EZ-2 evaporator to obtain a resinous propolis extract.

### 2.4. Preparative-Scale Isolation and Purification

The extract (202.1 mg) was fractionated using a C_18_ Kinetex HPLC column (5 μm, 250 × 21.2 mm) at a flow rate of 10 mL/min. The mobile phase consisted of H_2_O (solvent A) and MeCN (solvent B) running in 30 min with a linear gradient starting from 50% B to 80% B for 10 min and then increasing to 90% B for 20 min. In total, 30 fractions (1.0 min each) were collected. Fraction 14 contained pure compound **1** with a molecular weight (MW) of 424 Da (48.7 mg), and fractions 22–23 contained pure compound **5** (MW 492 Da, 9.2 mg). Fractions 12 and 13, consisting of the masses of interest (+) *m/z* 439 and (−) *m/z* 437, were combined and separated on the same C_18_ Kinetex HPLC column, with a linear gradient starting from 50% B to 75% B for 15 min, isocratic for 10 min and then increasing to 80% B for 5 min to obtain compound **6** (MW 438 Da, 5.0 mg, t_R_ = 15.0 min). Fraction 15, containing the masses of interest (+) *m/z* 425 and (−) *m/z* 423, was purified on the same C_18_ Kinetex HPLC column with a linear gradient starting from 55% B to 75% B for 5 min, isocratic for 15 min and then increasing to 80% B for 10 min to obtain compounds **1** (MW 424 Da, 6.0 mg, t_R_ = 12.0 min) and **2** (MW 424 Da, 6.2 mg, t_R_ = 14.0 min). Fraction 16 also containing the masses of interest (+) *m/z* 425 and (−) *m/z* 423 was purified on the same C_18_ Kinetex HPLC column, with a linear gradient starting from 55% B to 75% B for 5 min, isocratic for 5 min, and then increasing to 80% B for 10 min to obtain compounds **2** (MW 424 Da, 1.5 mg, t_R_ = 13.0 min) and **3** (MW 424 Da, 18.5 mg, t_R_ = 14.0 min). Fraction 17, containing the masses of interest (+) *m*/*z* 409 and (−) *m*/*z* 407, was purified on the same C18 Kinetex HPLC column, with a linear gradient starting from 55% B to 75% B for 5 min, isocratic for 15 min, and then increasing to 80% B for 10 min to obtain compound **4** (MW 408 Da, 5.4 mg, t_R_ = 16.0 min).

### 2.5. Analytical HPLC-DAD Analysis

The samples were analyzed on an analytical scale Agilent 1260 HPLC system with an injection volume of 20 μL. The separations were performed at room temperature on a Phenomenex SynergiTM 4 μm Fusion-RP 80 Å HPLC column (150 × 4.6 mm), with a flow rate of 1.0 mL/min. The mobile phase consisted of H_2_O (solvent A), and MeCN (solvent B). The samples were separated using a 20-min program, which was increased from 50% B to 60% B for 3.0 min, remained at 60% B for 8.0 min, increased to 80% B for 1 min, kept at 80% B for 3.0 min, increased to 100% B for 1.0 min, remained isocratic for 2.0 min, then reduced to 50% B for 0.5 min and finally re-equilibrated for 1.5 min. All chromatographic separations were controlled by Chemstation software.

### 2.6. LC–MS Analysis

All of the LC–MS analyses were performed using an analytical scale Agilent 1290 uHPLC system combined with an Agilent 6470 triple quadrupole mass spectrometer. The separations were performed at 35 °C on a Zorbax Eclipse Plus C18 column (50 × 2.1 mm, 1.8 μm particle size, 95 Å pore size) with a flow rate of 0.4 mL/min. The mobile phase consisted of H_2_O (solvent A), and MeCN (solvent B), both acidified with 0.1% formic acid. The samples were separated using a 15-min program which was started at 2% B for 0.5 min, increased to 100% B for 9.0 min, kept at this level for the next 3.0 min, then reduced to 2% B for 1 min and finally re-equilibrated for 1.5 min. The injection volume was 2 μL. The mass spectrometer was equipped with an ESI source. The mass spectra were acquired in both positive and negative ionization modes, using a gas temperature of 250 °C, a gas flow of 5 L/min, a capillary voltage of 4000 V, a nebulizer pressure of 30 PSI, a sheath gas heater of 400 °C, a sheath gas flow of 12 L/min, and a nozzle voltage of 1000 V. Chromatographic separation and mass spectrometry were controlled using the Mass Hunter software (B.09.00, Agilent Technologies, Santa Clara, CA, USA).

### 2.7. NMR Experiments

The NMR spectra were acquired on a Bruker Ascend 400 spectrometer equipped with a 5 mm room temperature probe operating at 400 MHz for ^1^H and 100 MHz for ^13^C. All of the experiments were acquired in automation (temperature equilibration to 298 K, optimization of lock parameters, gradient shimming, and setting of receiver gain). Compounds **1**, **2**, **3**, **4**, and **6** were dissolved in MeOH-*d*_4_, whereas compound **5** was dissolved in CHCl_3_-*d*. The ^1^H and ^13^C spectra were referenced to the residual deuterated solvent peaks at *δ*_H_ 3.31 and *δ*_C_ 49.0 (MeOH-*d*_4_), and *δ*_H_ 7.26 and *δ*_C_ 77.0 (CHCl_3_-*d*).

### 2.8. α-Glucosidase Inhibition Assay

The α-glucosidase inhibition assay was performed at room temperature in 96-well microtiter plates (Sigma Aldrich, Sydney, Australia). The assays were adapted from Zhao et al. [13], with some modifications. Briefly, in each well was added 10 μL of propolis sample at 2 mg/mL in DMSO and 90 μL of 0.1 M phosphate buffer, adjusted to pH 7.5 using 0.6 M KOH. To the solution, 80 μL of α-glucosidase solution (2.0 U/mL) was added and incubated for 10 min at room temperature. The enzyme reaction was initiated by adding 20 μL of p-NPG solution (10 mM in phosphate buffer). The colorimetric absorbance of the cleavage product (the strongly chromogenic p-nitrophenolate ion) after 30 min of incubation was measured at 405 nm using the EnSpire microplate reader (Perkin Elmer, Boston, MA, USA). Each experiment included one negative control (DMSO), and one positive control (acarbose) in parallel and were completed in triplicate. The results were analyzed and calculated in Microsoft Excel, and expressed using the mean for each of the triplicate sets. Inhibition of the enzyme was calculated as follows:

$$\mathrm{Inhibition}\;\left(\%\right)=\left(1-\frac{\mathrm{Absorbance}\;\left(\mathrm{sample}\right)}{\mathrm{Absorbance}\;\left(\mathrm{blank}\right)}\;\right)\times 100\%)$$

### 2.9. α-Amylase Inhibition Assay

The α-amylase inhibition assay was performed in 96-well microtiter plates. In each well was added 10 μL of propolis sample dissolved in DMSO, and 90 μL of 0.1 M phosphate buffer adjusted to pH 6.0 using 2.0 M H_3_PO_4_. To the solution, 80 μL of human salivary α-amylase solution (2.0 U/mL) was added and incubated for approximately 10 min at 37 °C. The enzyme reaction was initiated by adding 20 μL of the substrate solution CNP-G3 (10 mM in phosphate buffer). The colorimetric absorbance after 120 min of incubation was measured at 405 nm using the EnSpire microplate reader (Perkin Elmer). Each experiment included one negative control (DMSO), and one positive control (acarbose) in parallel and were completed in triplicate. The percentage inhibition was calculated using the same formula as described for the α-glucosidase inhibition assay.

### 2.10. Lipase Inhibition Assay

The lipase inhibition assay was performed in 96-well microtiter plates. In each well was added 10 μL of propolis sample dissolved in DMSO and 155 μL of 0.1 M tris-HCl buffer adjusted to pH 7.4 using 1.0 M KOH. To the solution, 25 μL of lipase solution (2000 U/mL) was added and incubated for approximately 15 min at 37 °C. The enzyme reaction was initiated by adding 10 μL of the substrate solution p-NPB (100 mM in methanol). The colorimetric absorbance of the cleavage product after 60 min of incubation was measured at 405 nm, using the EnSpire microplate reader (Perkin Elmer). Each experiment included one negative control (DMSO), and one positive control (orlistat) in parallel and was completed in triplicate. The percentage inhibition was calculated using the same formula as described for the α-glucosidase inhibition assay.

### 2.11. α-Glucosidase Binding Assay

At a concentration of 6.25 mg/mL, 10 μL of sample extract was incubated at room temperature with 40 μL of the α-glucosidase enzyme (10.0 U/mL) in 150 μL of 0.1 M phosphate buffer (pH 7.5) for 60 min in darkness. After incubation, the mixture was filtered through a 10 kDa Microcon^®^ centrifugal filter device (Merck), and centrifuged at 14,000× *g* for 10 min. After centrifugation, the filter was washed three times to remove unbound compounds using 100 μL aliquots of 0.1 M phosphate buffer (pH 7.5) and was centrifuged at 14,000× *g* for 10 min after each wash. The bound ligands were released by adding 100 μL of acetonitrile to denature the enzyme, followed by centrifugation at 14,000× *g* for 10 min; this step was repeated twice. The supernatants were combined and used for LC–MS analysis.

### 2.12. Molecular Docking Study

The Protein Data Bank structure of α-glucosidase from *Bacteroides thetaiotaomicron* (PDB code: 2ZQ0), human pancreatic α-amylase (PDB code: 2QV4), and the lipase from *Staphylococcus aureus* (PDB code: 6KSM) were downloaded from the Research Collaboratory for Structural Bioinformatics (RCSB) Protein Data Bank. The protein structures were analyzed using Discovery Studio 4.5. The water molecules, heteroatoms, and ligands were removed, and the polar hydrogen atoms were added to the structures. Depending on the binding mode of the co-crystalized ligands, the active site residues of the proteins were determined using Discovery Studio 4.5 and, then, the binding site sphere was defined accordingly. In the α-glucosidase, α-amylase, and lipase proteins, the dimension of the sphere was 20 Å, and the center (x, y, z) of the sphere was (27.776172, 56.165741, 35.362259), (12.384745, 48.136073, 26.209218), and (26.571972, 33.131000, −11.545972), respectively. The docking calculation was subsequently performed using AutoDock Vina. The top nine binding poses were opted for prediction and results were analyzed, using Discovery studio visualizer. The docking study was validated by redocking and superimposing the co-crystallized ligand (acarbose or lipase) with extracted ligand from the crystal structure. The calculated root-mean-square deviation (RMSD) values of acarbose and orlistat were all less than 2 Å.

## 3. Results and Discussion

The propolis extract collected in Queensland, Australia inhibited α-glucosidase by 42% at a concentration of 100 μg/mL and displayed an IC_50_ value of 118.1 μg/mL in an in vitro assay. Compared to a positive control acarbose with an IC_50_ value of 256.1 μg/mL, this propolis showed two-fold more potency. The results indicated the potential anti-diabetes effect of this Australian propolis and therefore it would be valuable to identify potent α-glucosidase inhibitors from this source.

### 3.1. α-Glucosidase Binding Assay

Compared to a conventional bioactivity-guided fractionation and purification method, an affinity-based ligand fishing demonstrates more time effectiveness and selectivity for screening potential enzyme inhibitors in complex natural product mixtures, including extracts or fractions [31]. Among some of the techniques that have been used successfully to fish enzyme inhibitors, such as ultrafiltration [32,33,34], immobilized silica gel [35], immobilized magnetic microspheres [3,36,37], and surface plasmon resonance [38], the affinity ultrafiltration method has been proven to be more convenient and cost-effective [31]. The principle of this method is based on the formation of ligand–enzyme complexes, after the enzyme is incubated with an extract having enzyme inhibitors. These complexes are trapped, and unbound compounds are eluted from the mixture by ultrafiltration. The ligand–enzyme complexes retained on the membrane are disrupted by the addition of organic solvent to release small molecular ligands which are then identified by either HPLC-UV or LC–MS [31].

**Figure 1:**
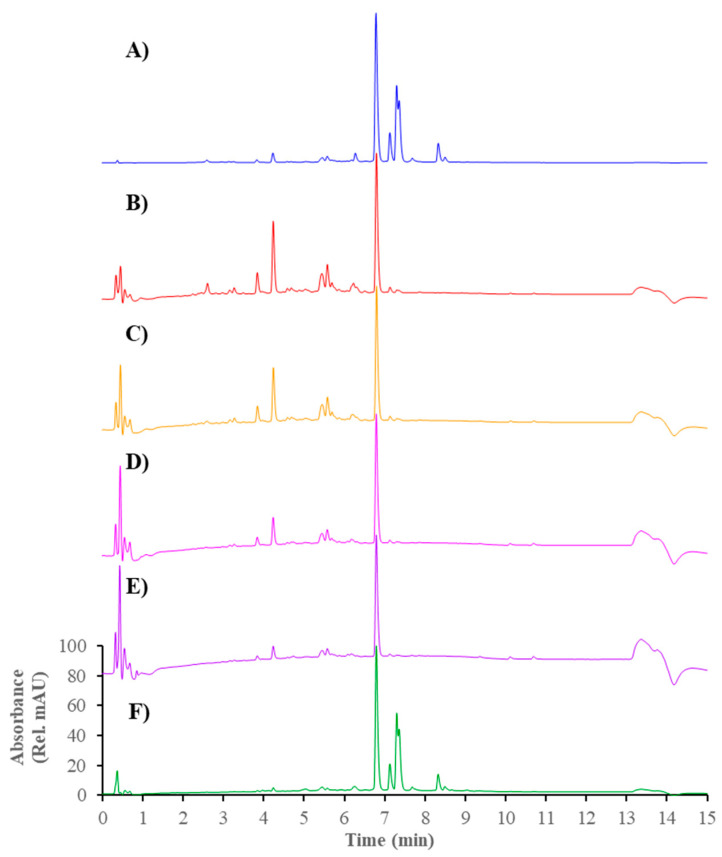
HPLC-UV profiles (λ = 280 nm) presenting bound and unbound compounds of propolis extract to α-glucosidase. (**A**) Full extract; (**B**) Filtrate; (**C**) Supernatant after 1st washing; (**D**) Supernatant after 2nd washing; (**E**) Supernatant after 3nd washing; and (**F**) Final supernatant.

The active propolis extract found from the in vitro α-glucosidase inhibitory assay was further subjected to a binding assay, using the affinity ultrafiltration method to identify the α-glucosidase inhibitors. The HPLC-UV profiles of the unbound and bound compounds in the propolis are shown in Figure 1. The initial two profiles (blue and red) display all of the compounds in the propolis extract, and the unbound compounds (non-binding ligands) after incubation with α-glucosidase. The subsequent three profiles (yellow, pink, and purple) show the unbound compounds present in the filtrates after the ligand–enzyme complexes were washed with phosphate buffer to ensure no remaining unbound compounds. The final chromatogram (green) shows the ligands bound to α-glucosidase after they were released from the enzyme. It should be noticed that a peak at 6.793 min was present in all of the chromatograms, which indicated a large excess of this compound over the α-glucosidase enzyme. From the 2 μL injection, at least eight ligands with corresponding molecular weights predicted from MS data were found from this experiment (Figure 1, F and Table 1).

### 3.2. NMR Identification of α-Glucosidase Inhibitors

Mass-guided fractionation was employed to isolate the α-glucosidase ligands detected from the ultrafiltration LC–MS binding assay (Table 1). Six out of eight of the compounds were successfully purified from the propolis extract. The interpretation of their NMR and MS data (Figures S1–S30, Supplementary Materials) allowed for the identification of five known prenylated flavanones, including propolin D (Nymphaeol B) (**1**) [39], propolin F (Isonymphaeol B) (**2**) [40], propolin C (Nymphaeol A) (**3**) [41], propolin H (3′-geranyl-naringenin) (**4**) [39], and propolin G (Nymphaeol C) (**5**) [39]; and one known prenylated flavonol, solophenol D (**6**) (Figure 2) [42].

### 3.3. Inhibitory Activity Assessment of the Isolated Compounds

The inhibitory activity of the six isolated compounds against α-glucosidase was confirmed by an enzyme assay. Their α-amylase and lipase inhibition capacity was also examined. The precursors of the isolated compounds, including eriodictyol (**7**), naringenin (**8**), and quercetin (**9**) were included in these assays for the investigation of their structure activity relationships (SARs).

Compounds **1**, **2**, **4**, **6,** and **9** inhibited α-glucosidase with IC_50_ values in a range of 19.2–298.4 µM, which was more potent than the positive control acarbose (IC_50_ = 396.7 µM) (Table 2). All of the flavanone compounds (**1**–**5** and **7**–**8**) were less active than the flavonols (**6** and **9**) up to 40-folds (**5** versus **9**). Compound **9** was the most potent α-glucosidase inhibitor. Its inhibitory activity reduced when H-2′ of the flavonol scaffold was replaced by the geranyl group in compound **6**. Among all tested flavanones, compound **4** exhibited the highest activity with an IC_50_ value of 178.5 µM. Comparing the α-glucosidase inhibition of compounds **4**, **8**, **2** and **7** indicated that the presence of the hydroxy group at C-3′ decreased the activity while the geranyl group at C-5′ of the flavanone did not impact significantly the α-glucosidase inhibition. The results of the compounds **1**–**5**, **7,** and **8** revealed that adding the geranyl group to the flavanone scaffold enhanced the α-glucosidase inhibition, whereas adding the prenyl group at C-6 (compound **5**) decreased the activity. This decrease may be due to an increase in a molecular size, which prevents the molecule from entering a binding site leading to reducing its interaction with the enzyme.

**Figure 3:**
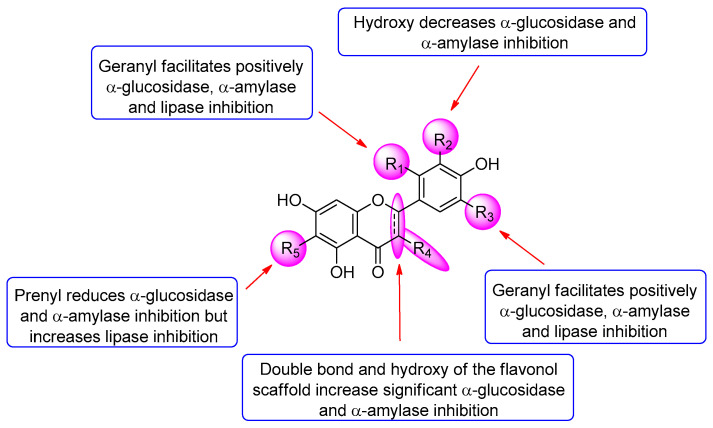
Structure activity relationship of flavonoids in this study.

In terms of the α-amylase inhibition, all of the compounds were more active than acarbose and exhibited a stronger inhibitory effect against α-amylase than α-glucosidase (Table 2). In general, all of the compounds displayed similar SAR patterns in the α-amylase inhibition compared to the α-glucosidase inhibition (Figure 3). Regarding the lipase inhibition, all of the nine compounds showed 6–10-fold less activity than a positive control orlistat, but much more potent than α-glucosidase and α-amylase inhibition (Table 2). A comparison of the lipase inhibition of the six isolated compounds (**1**–**6**) versus their precursors (**7**–**9**) indicated that adding the geranyl and prenyl groups to both the flavonols and flavanones dramatically increased inhibitory activity. This result revealed that the hydrophobicity of the flavonoids might have a significant effect on the lipase inhibition. However, no significant difference in the SAR between the six isolated compounds was observed.

Owing to the prenylated side chains, which can enhance cell membrane permeability, the prenylated flavonoids generally show stronger biological activities than their non-prenylated precursors [43,44]. They have been known to possess a wide range of biological properties, such as antifungal [45], antimicrobial [46,47], antiviral [48,49], anti-inflammatory [50], anticancer [47,51,52], anti-arthritic [50], anti-osteoporosis [52,53], anti-lipid [54], and α-glucosidase inhibitory activities [55], in both in vitro and *in vivo* studies. More recently, the synergistic effects of some of the prenylated flavonoids in combination with conventional antibiotics, including vancomycin, ciprofloxacin, and methicillin, against the multidrug-resistant *Staphylococcus aureus* bacterial strain have been reported [56]. Due to their beneficial effects on human health, the prenylated flavonoids are of interest as the lead compounds for the development of drugs and functional foods [57]. Although the antioxidant, antibacterial, anti-inflammatory, anticancer, anti-Alzheimer’s disease, and α-glucosidase inhibitory activities of the compounds **1**–**5** in this study were previously reported (Table 3), this is the first time that their α-amylase and lipase inhibitory activities have been assessed. The understanding of SAR in this study will help facilitate the development of prenylated flavonoid-based nutraceuticals to prevent diabetes and obesity diseases in the future.

### 3.4. Ligand–Protein Interactions

A molecular docking study was performed to enhance the basic knowledge of how the isolated compounds inhibited the targeted enzymes. Two parameters, affinity energy (kcal/mol) and the number of interactions, were used to select the most favored pose and compare the ligand–protein interactions. In principle, a lower affinity energy corresponds to better stability of the inhibitory system, while more bonding interactions formed between the ligand and its protein target indicate a higher possibility that the molecule binds to the protein [66].

**Figure 4:**
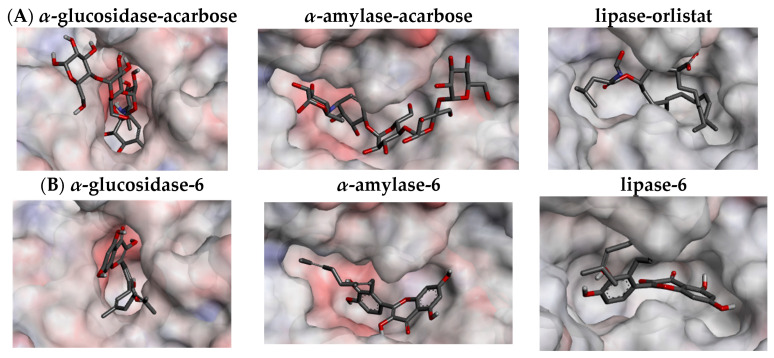
Binding pocket of *α*-glucosidase (2ZQ0), *α*-amylase (2QV4), and lipase (6KSM) with their ligands (enzyme surface shows interpolated charge with positive in blue, zero in white, and negative in red). (**A**) Positive controls; and (**B**) Compound **6**.

The docking study confirmed that all of the flavonoids and their precursors interacted with α-glucosidase and α-amylase on the acarbose-binding site. The six isolated compounds (**1**–**6**) showed the affinity energy ranging from −9.8 to −9.2 kcal/mol in α-glucosidase, and from −10.4 to −9.4 kcal/mol in α-amylase which were comparable to the positive control, acarbose (**10**) (Table 4). However, the affinity energy of their precursors (**7**–**9**) was noticeably higher. The three-dimensional enzyme structures indicated that the binding pocket of α-glucosidase is less capacious than that of α-amylase, which possibly limits its ligands to enter an active site (Figure 4A). This might be a reason why the flavonoids in the in vitro assays displayed more inhibitory activity against α-amylase than α-glucosidase. While acarbose almost interacted with α-glucosidase and α-amylase via hydrogen bonds, the flavonoids exhibited diverse interactions, including hydrogen, π-π, π-σ, π-alkyl, alkyl, and electrostatic bonds (Table 4 and Figures S31–S32, Supplementary Materials). With the extension of the π-π conjugation system, the flavonol scaffold (**6** and **9**) formed more hydrophobic π-π and π-alkyl interactions and therefore bound more strongly to the enzymes than the flavanone scaffold (**1**–**5** and **7**–**8**). Moreover, the docking data showed that the addition of the geranyl group to the flavonoid molecules enhanced the number of binding interactions and affinity energy, suggesting an increase in their inhibitory activity. The conflicting results between in silico and in vitro assays might be due to the fact that the docking study did not consider the solubility of the compounds in an assay buffer.

An in-pose configuration of the lipase–orlistat complex was visually projected in Figure 4A. In general, the binding pocket of lipase is relatively larger than that of the α-glucosidase, but smaller than that of α-amylase. This active site also has less interpolated charges, which indicates that it possibly interacts with the ligands mainly via the hydrophobic bonds. The docking study indicated all of the flavonoid compounds fitted the lipase binding pocket. Unlike the binding of orlistat (**11**), which depends on the hydrophobic π-alkyl and alkyl bonds, the driving force of the interactions between the flavonoids and lipase is a mixture of hydrogen bonds, π-σ, π-π, π-alkyl, and alkyl (Table 4). Surprisingly, all of the tested flavonoids showed lower affinity energy (−10.1 to −8.1 kcal/mol) than the positive control, orlistat (**11**) (−7.0 kcal/mol). Similar to the α-glucosidase and α-amylase cases, the presence of the geranyl group increased the number of hydrophobic π-alkyl bonds for compounds **1**–**6**, resulting in their stronger interactions with the lipase compared to their precursors (**7**–**9**). Therefore, the in silico assay further supported the in vitro activity of the compounds **1**–**6** against the lipase enzyme.

### 3.5. Propolis Identification

**Figure 5:**
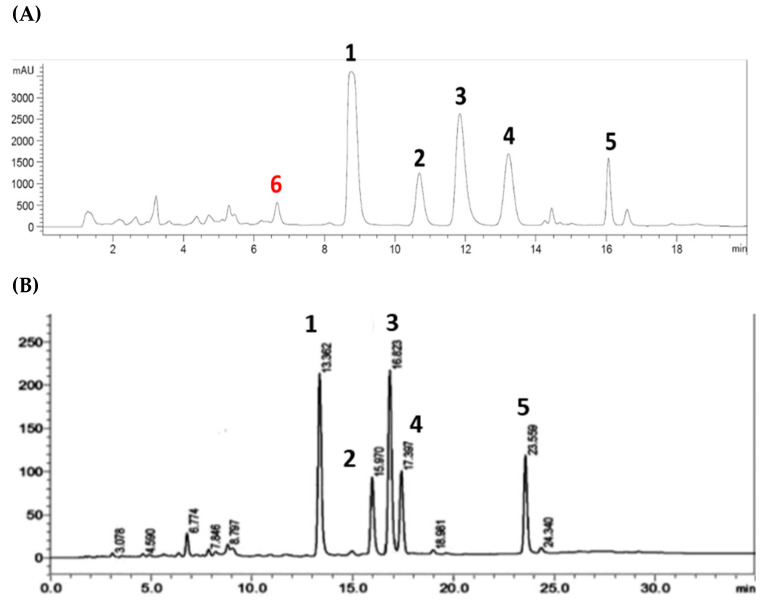
HPLC chromatograms of the Australian propolis extract in this study (**A**) and Taiwanese green propolis extract (**B**) (adapted from Chen et al. [67]) recorded at 280 nm.

Of the six compounds isolated from the Australian propolis, five were discovered as chemical markers of the propolis found in Pacific regions, including Okinawa (South of Japan), Taiwan, and the Solomon Islands. A comparison between the HPLC profiles of the propolis extract in this study and the one reported by Chen et al. [67] (Figure 5) indicates that this Australian propolis shares a relatively similar chemical fingerprint to the Taiwanese propolis, with five major components (**1**–**5**). However, solophenol D (**6**) is potentially a unique chemical marker of the Australian propolis. So far, compound **6** has only been isolated from the Solomon Island propolis [42], and has not been reported in the other Pacific propolis collected in Okinawa and Taiwan.

It was known that the Taiwanese and Okinawan propolis originated from the *Macaranga tanarius* fruit resin [68], while the botanical origin of the Solomon Island propolis has not been identified. This study showed that the chemical composition of the Queensland propolis was more similar to the Solomon Islands propolis, suggesting that they might come from a similar botanical source, which may or may not be the *Macaranga tanarius* resin. Further search on the original resin and the distribution of this propolis type in Australia will warrant an in-depth understanding of this bioactive source.

## 4. Conclusions

In summary, from the chemical investigation of the bioactive Australian propolis identified from the α-glucosidase inhibitory screening, this study found a new Australian propolis type with a chemical composition relatively close to the Pacific propolis type. The purified prenylated flavonoids displayed potent α-glucosidase, α-amylase, and lipase inhibitory activities on both in vitro and in silico assays. Currently, Okinawan and Taiwanese propolis are commercialized as herbal remedies for safe, daily intake, and in the development of functional foods [69]. Therefore, the Australian propolis type in this study will potentially become a valuable source for the development of functional foods to prevent hyperglycemic complications, such as type 2 diabetes mellitus, pre-diabetes, and obesity.

## Figures and Tables

**Figure 2:**
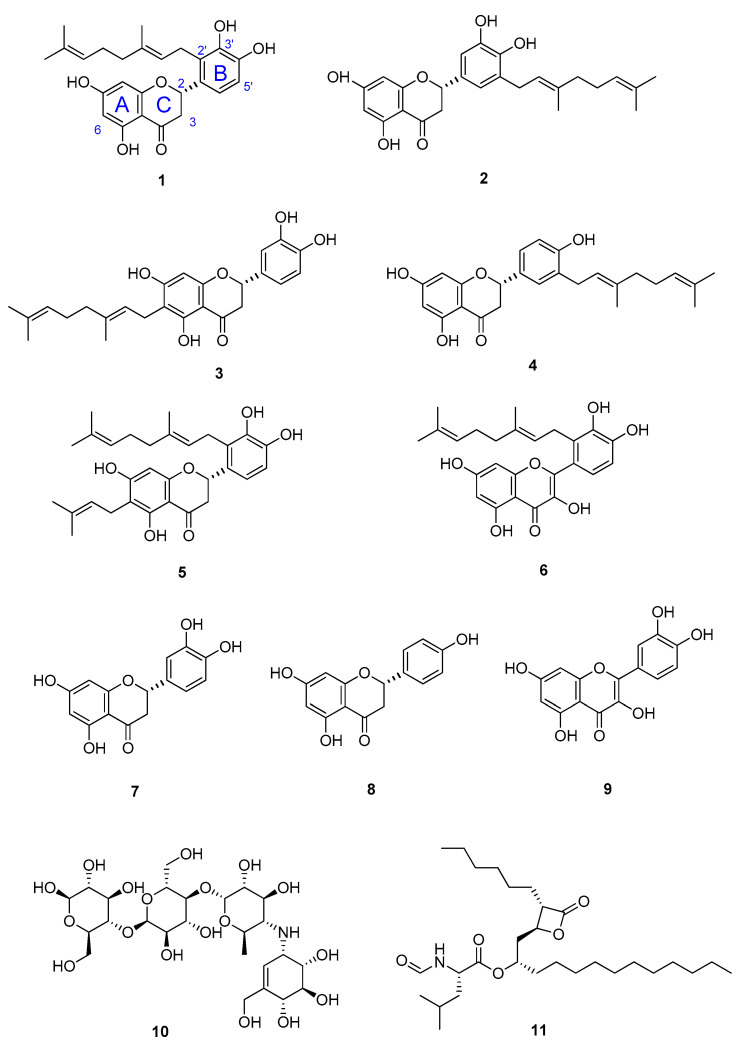
Chemical structures of compounds **1**–**6** isolated from the Australian propolis, their precursors eriodictyol (**7**); naringenin (**8**); and quercetin (**9**); antidiabetic drug, acarbose (**10**); and anti-obesity drug, orlistat (**11**).

**Table 1:** Predicted molecular weight (MW) of the α-glucosidase ligands.

Retention Time (min)	(+) *m/z* [M+H]^+^	(−) *m/z* [M-H]^−^	Predicted MW
6.267	439	437	438
6.793	425	423	424
7.133	425	423	424
7.300	425	423	424
7.360	409	407	408
7.687	409	407	408
8.327	493	491	492
8.493	493	491	492

**Table 2:** α-Glucosidase, α-amylase and lipase inhibition of compounds **1**–**9**.

Compound	IC_50_ (µM)		
	*α*-Glucosidase	*α*-Amylase	Lipase
**1**	252.4	204.6	51.8
**2**	298.4	134.9	53.0
**3**	421.3	203.9	30.3
**4**	178.5	52.5	33.1
**5**	776.6	246.0	43.4
**6**	57.8	142.1	32.3
**7**	13% at 250 µM	41% at 250 µM	12% at 83 µM
**8**	647.4 *^c^*	121.5 *^d^*	24% at 83 µM
**9**	19.2	17.4	37% at 83 µM
**Acarbose *^a^***	396.7	624.7	- *^b^*
**Orlistat *^a^***	- *^b^*	- *^b^*	5.4

*^a^* Positive control; *^b^* Not determined; *^c^* 40% at 250 µM; *^d^* 73% at 250 µM.

**Table 3:** An overview of the biological properties of the six compounds isolated from the Queensland propolis.

Compounds	Biological Properties
Propolin D (**1**)	Antioxidant [39], antimicrobial [58,59], anti-inflammatory [60], anti-Alzheimer’s [60], anticancer [61], *α*-glucosidase inhibition [60], *α*-amylase inhibition *, lipase inhibition *
Propolin F (**2**)	Antioxidant [39], antimicrobial [58,59], anti-inflammatory [60], anti-Alzheimer’s [60], anticancer [61], *α*-glucosidase inhibition [60], *α*-amylase inhibition *, lipase inhibition *
Propolin C (**3**)	Antioxidant [39], antimicrobial [58,59], anti-inflammatory [60], anti-Alzheimer’s [60], anticancer [41,61], *α*-glucosidase inhibition [60], *α*-amylase inhibition *, lipase inhibition *
Propolin H (**4**)	Antioxidant [39], antimicrobial [59], anti-inflammatory [60], anti-Alzheimer’s [60], anticancer [62], *α*-glucosidase inhibition [60], *α*-amylase inhibition *, lipase inhibition *
Propolin G (**5**)	Antioxidant [39], antimicrobial [58,59], anti-inflammatory [60,63], anti-Alzheimer’s [60], anticancer [64], hepatoprotective [65], *α*-glucosidase inhibition [60], *α*-amylase inhibition *, lipase inhibition *
Solophenol D (**6**)	Antibacterial [42], *α*-glucosidase inhibition *, *α*-amylase inhibition *, lipase inhibition *

* Biological properties were found in this study.

**Table 4:** Binding affinity and binding interactions of compounds **1**–**11** *^a^* to *α*-glucosidase, *α*-amylase and lipase enzyme.

Compound	Binding Affinity (kcal/mol)	Hydrogen Bond (Distance, Å)	Hydrophobic Bond (Distance, Å)	Electrostatic Bond (Distance, Å)	Total Interactions
***α*-glucosidase** **(2ZQ0)**					
**1**	−9.4	Met^334^ (1.93); Glu^508^ (1.90); Glu^532^ (2.77)	**π-π:** Phe^536^ (4.89) **π-alkyl:** Tyr^533^ (5.02); Phe^536^ (4.46); Phe^536^ (4.83); Val^471^ (4.80)	Glu^532^ (3.91)	9
**2**	−9.3	Asn^216^ (2.55); His^437^ (3.09); Ser^217^ (1.55); Glu^391^ (2.81)	**π-alkyl:** Phe^536^ (5.02); Phe^536^ (4.66); Phe^536^ (4.83); Val^471^ (5.18); Val^471^ (5.48)	Glu^439^ (3.99)	10
**3**	−9.8	Glu^508^ (2.59); Glu^391^ (1.89); Trp^341^ (2.41)	**π-π:** Phe^536^ (4.24) **π-alkyl:** Phe^536^ (4.44); Val^471^ (5.24)		6
**4**	−9.3	Ser^217^ (2.31); Glu^532^ (2.16)	**π-σ:** Val^471^ (3.74); Phe^536^ (3.82) **π-π:** Trp^341^ (5.41); Phe^401^ (5.21) **π-alkyl:** Phe^536^ (4.03)		7
**5**	−9.6	Glu^508^ (2.09); Glu^508^ (2.79); Glu^194^ (3.08); Met^334^ (2.07)	**π-π:** Phe^536^ (4.85) **π-alkyl:** Tyr^533^ (5.02); Phe^536^ (4.47); Phe^536^ (4.19); Val^471^ (4.94)	Glu^532^ (3.80)	10
**6**	−9.2	Ser^217^ (1.88); Ser^217^ (2.60); Ser^217^ (2.07); Ser^217^ (2.62); Glu^194^ (3.06)	**π-σ:** Val^471^ (3.91) **π-alkyl:** Trp^341^ (5.45); Trp^341^ (4.93); Trp^400^ (5.09); Phe^401^ (4.84); Phe^401^ (4.19); Phe^536^ (4.64) **Alkyl:** Ile^335^ (4.61)	Glu^532^ (3.84)	14
**7**	−8.9	Lys^467^ (2.64); Glu^391^ (2.61); Ser^217^ (1.11)	**π-alkyl:** Val^471^ (5.49)	Glu^439^ (4.47) Glu^532^ (4.17)	6
**8**	−8.4	Ser^217^ (2.28); Glu^194^ (2.65)	**π-π:** Trp^341^ (4.76) **π-alkyl:** Val^471^ (4.35)		4
**9**	−8.4	Ser^217^ (2.43); Trp^331^ (2.12); Trp^331^ (2.30); Glu^391^ (2.12); Asn^216^ (2.87); His^437^ (3.42)	**π-π:** Trp^341^ (5.47); Trp^400^ (5.53) **π-alkyl:** Val^471^ (5.17); Val^471^ (5.32)		10
**Acarbose** **(10) *^b^***	−9.7	Asn^216^ (2.86); Trp^331^ (2.01); Trp^341^ (2.67); His^507^ (2.89); Glu^532^ (3.06); Glu^532^ (2.40); Glu^508^ (2.11); Glu^526^ (2.37); Glu^391^ (2.33); His^437^ (2.34); Pro^215^ (2.24); Pro^215^ (2.35); Pro^215^ (3.71); Phe^536^ (3.42); Phe^536^ (2.66); Ser^217^ (2.00)	**π-alkyl:** Phe^536^ (5.31)		17
***α*-Amylase** **(2QV4)**					
**1**	−10.4	Gln^63^ (2.69); Asp^197^ (3.02); His^299^ (2.45)	**π-π:** Trp^59^ (3.84); Trp^59^ (4.21); Tyr^62^ (4.30) **π-alkyl:** His^201^ (4.93) **Alkyl:** Leu^162^ (4.11); Leu^162^ (4.39); Lys^200^ (4.49); Ile^235^ (4.64)	Asp^300^ (3.79)	12
**2**	−9.5	Gln^63^ (2.47); Gln^63^ (1.33); Asp^197^ (2.36); His^299^ (2.34); Glu^233^ (2.68)	**π-π:** Trp^59^ (4.16); Trp^59^ (4.14); Tyr^62^ (4.67) **Alkyl:** Leu^162^ (5.41); Ala^198^ (4.89)	Asp^197^ (4.94)	11
**3**	−10.0	Gln^63^ (2.56); Asp^300^ (2.05); Asp^197^ (2.95)	**π-π:** Trp^59^ (4.22); Trp^59^ (4.22); Tyr^62^ (4.22) **π-alkyl:** Trp^59^ (4.49) **Alkyl:** Ile^51^ (3.77); Val^107^ (3.97)		9
**4**	−9.5	Gln^63^ (2.55); His^299^ (2.84); Asp^300^ (2.21)	**π-π:** Trp^59^ (3.86); Trp^59^ (4.09); Tyr^62^ (4.45) **Alkyl:** Leu^162^ (5.48); Ala^198^ (4.90)		8
**5**	−9.4	His^305^ (2.62); His^201^ (2.41); His^305^ (3.24)	**π-σ:** Tyr^62^ (3.74); Trp^59^ (3.82) **π-alkyl:** Trp^59^ (3.95); Trp^59^ (4.71); Trp^59^ (4.34); Tyr^62^ (5.32); Leu^162^ (4.89); Ile^235^ (5.33) **Alkyl:** Ala^198^ (5.23)		12
**6**	−10.0	Gln^63^ (1.99); Asp^300^ (2.95); Asp^197^ (2.38); Arg^195^(2.13)	**π-π:** Trp^59^ (4.98); Trp^59^ (4.65); Trp^59^ (4.60); Tyr^62^ (4.48) **π-alkyl:** His^201^ (4.97) **Alkyl:** Leu^162^ (4.25); Lys^200^ (4.40); Ile^235^ (4.37)	Asp^300^ (3.71)	13
**7**	−9.0	Gln^63^ (2.47); Gln^63^ (2.27); His^299^ (2.75); Asp^300^ (2.48); His^305^ (3.65)	**π-π:** Trp^59^ (3.86); Trp^59^ (4.12); Tyr^62^ (4.27)		8
**8**	−8.9	Gln^63^ (2.39); Asp^197^ (2.02); His^305^ (3.62); Arg^195^(2.23)	**π-π:** Trp^59^ (3.86); Trp^59^ (4.10); Tyr^62^ (4.11)		7
**9**	−9.0	Gln^63^ (2.20); Gln^63^ (2.61); Asp^300^ (3.06); Tyr^62^ (2.74); His^305^ (3.58)	**π-π:** Trp^59^ (5.09); Trp^59^ (3.87); Trp^59^ (5.40); Trp^59^ (4.03); Tyr^62^ (4.54)		10
**Acarbose (10) *^b^***	−10.1	Gln^63^ (1.97); Asn^105^ (2.50); Asn^105^ (2.63); Ala^106^ (1.94); Thr^163^ (2.85); Arg^195^(2.23); His^305^ (2.76); Glu^233^ (3.29); Asp^300^ (2.91); Gly^164^ (3.39); Thr^163^ (3.56); Glu^233^ (3.61); His^305^ (3.83); Tyr^62^ (3.50)			14
**Lipase** **(6KSM)**					
**1**	−9.4	Phe^17^ (2.32); Phe^17^ (2.31); Phe^17^ (2.92); Ala^175^ (2.28)	**π-σ:** Val^309^ (3.87) **π-alkyl:** Phe^17^ (5.41); Phe^59^ (4.93); Phe^178^ (5.24); Ala^174^ (4.81); Ala^175^ (5.41) **Alkyl:** Leu^18^ (5.33); Leu^18^ (4.12); Leu^18^ (4.49); Met^188^ (4.34)		14
**2**	−10.1	His^115^ (2.64); Tyr^32^ (2.28); Tyr^32^ (2.87); Ser^172^ (3.12)	**π-π:** His^349^ (4.64) **π-alkyl:** Tyr^32^ (4.56); Phe^285^(4.91); Ala^174^ (5.04); Ala^175^ (4.90); Leu^242^ (4.22); Val^309^ (4.81) **Alkyl:** Pro^30^ (4.73); Pro^30^ (4.25); Val^350^ (4.63); Val^355^ (3.86); Val^355^ (4.62); Leu^287^ (4.30); Ile^353^ (5.45)		18
**3**	−9.2	Ala^175^ (2.90); Leu^242^ (2.74); Tyr^32^ (2.60); Ser^172^ (2.96)	**π-π:** His^349^ (5.91) **π-alkyl:** Phe^285^(4.75); Leu^18^ (5.37); Pro^168^ (4.98); Leu^242^ (4.39); Val^309^ (4.60); Val^310^ (5.48) **Alkyl:** Pro^30^ (5.32); Val^350^ (5.01); Val^355^ (4.94); Leu^287^ (5.29)		15
**4**	−9.5		**π-σ:** Val^309^ (3.92) **π-alkyl:** Phe^17^ (5.36); Phe^17^ (4.65); Phe^178^ (5.41); Leu^242^ (5.20); Met^288^ (5.27); Val^350^ (5.03) **Alkyl:** Ala^174^ (4.69); Ala^175^ (3.98); Ala^175^ (3.79); Ala^239^ (4.33); Leu^242^ (4.90); Leu^242^ (4.73); Val^309^ (5.29)		14
**5**	−10.0	Phe^17^ (2.29); Phe^17^ (2.84); Phe^17^ (2.90); Ala^175^ (2.35)	**π-alkyl:** Phe^17^ (5.32); Tyr^29^ (4.46); Phe^59^ (5.28); Phe^178^ (5.35); Ala^174^ (5.06); Ala^175^ (5.30); Leu^242^ (4.56); Val^309^ (4.47) **Alkyl:** Leu^18^ (5.21); Leu^18^ (4.35); Met^188^ (4.25); Pro^30^ (4.76)		16
**6**	−9.1		**π-σ:** Val^309^ (3.72); Val^309^ (3.94) **π-π:** Phe^17^ (5.27); His^349^ (4.35); His^349^ (5.68) **π-alkyl:** Ala^174^ (4.75); Ala^175^ (5.23); Val^309^ (5.07) **Alkyl:** Met^288^ (4.61); Val^350^ (5.34); Val^350^ (5.24)		11
**7**	−8.6	Gly^16^ (3.07); Leu^242^ (2.87)	**π-π:** His^349^ (4.45) **π-alkyl:** Ala^174^ (5.03); Ala^175^ (4.88); Leu^242^ (4.21); Val^309^ (4.82)		7
**8**	−8.1		**π-π:** Phe^17^ (5.59) **π-alkyl:** Ala^174^ (4.90); Ala^175^ (4.91); Leu^242^ (4.55); Val^309^ (4.83)		5
**9**	−8.6	Phe^17^ (2.92); Ala^175^ (2.74); Tyr^32^ (2.53)	**π-σ:** Val^309^ (3.86) **π-π:** His^349^ (4.36); His^349^ (5.69) **π-alkyl:** Ala^174^ (4.73); Ala^175^ (5.24); Val^309^ (5.04)		9
**Orlistat (11) *^b^***	−7.0	His^115^ (3.71); His^349^ (3.41)	**π-alkyl:** Phe^17^ (5.40); Phe^17^ (5.48); Tyr^32^ (4.86); Phe^178^ (5.00); His^349^ (4.72) **Alkyl:** Leu^18^ (5.46); Leu^18^ (4.42); Pro^168^ (4.22); Ala^174^ (4.27); Ala^175^ (3.94); Val^309^ (4.73); Val^309^ (5.34); Val^310^ (5.12); Val^355^ (5.35); Met^188^ (5.10); Leu^242^ (4.08); Leu^242^ (4.64)		19

*^a^* The best docked pose; *^b^* Positive control.

## Data Availability

The data generated for this study are available in the Supplementary Materials.

## Supplementary Materials

The following supporting information can be downloaded at: https://www.mdpi.com/article/10.3390/foods11131964/s1. Figure S1: ^1^H Spectrum of **1** in MeOH-*d_4_*; Figure S2: ^13^C Spectrum of **1** in MeOH-*d_4_*; Figure S3: HSQC Spectrum of **1** in MeOH-*d_4_*; Figure S4: gCOSY Spectrum of **1** in MeOH-*d_4_*; Figure S5: HMBC Spectrum of **1** in MeOH-*d_4_*; Figure S6: ^1^H Spectrum of **2** in MeOH-*d_4_*; Figure S7: ^13^C Spectrum of **2** in MeOH-*d_4_*; Figure S8: HSQC Spectrum of **2** in MeOH-*d_4_*; Figure S9: gCOSY Spectrum of **2** in MeOH-*d_4_*; Figure S10: HMBC Spectrum of **2** in MeOH-*d_4_*; Figure S11: ^1^H Spectrum of **3** in MeOH-*d_4_*; Figure S12: ^13^C Spectrum of **3** in MeOH-*d_4_*; Figure S13: HSQC Spectrum of **3** in MeOH-*d_4_*; Figure S14: gCOSY Spectrum of **3** in MeOH-*d_4_*; Figure S15: HMBC Spectrum of **3** in MeOH-*d_4_*; Figure S16: ^1^H Spectrum of **4** in MeOH-*d_4_*; Figure S17: ^13^C Spectrum of **4** in MeOH-*d_4_*; Figure S18: HSQC Spectrum of **4** in MeOH-*d_4_*; Figure S19: gCOSY Spectrum of **4** in MeOH-*d_4_*; Figure S20: HMBC Spectrum of **4** in MeOH-*d_4_*; Figure S21: ^1^H Spectrum of **5** in CHCl_3_-*d*; Figure S22: ^13^C Spectrum of **5** in CHCl_3_-*d*; Figure S23: HSQC Spectrum of **5** in CHCl_3_-*d*; Figure S24: gCOSY Spectrum of **5** in CHCl_3_-*d*; Figure S25: HMBC Spectrum of **5** in CHCl_3_-*d*; Figure S26: ^1^H Spectrum of **6** in MeOH-*d_4_*; Figure S27: ^13^C Spectrum of **6** in MeOH-*d_4_*; Figure S28: HSQC Spectrum of **6** in MeOH-*d_4_*; Figure S29: gCOSY Spectrum of **6** in MeOH-*d_4_*; Figure S30: HMBC Spectrum of **6** in MeOH-*d_4_*; Figure S31: Two-dimensional diagrams showing non-covalent interactions of **1**–**9** and acarbose (**10**) with α-glucosidase (2ZQ0); Figure S32: Two-dimensional diagrams showing non-covalent interactions of **1**–**9** and acarbose (**10**) with α-amylase (2QV4); Figure S33: Two-dimensional diagrams showing non-covalent interactions of **1**–**9** and orlistat (**11**) with lipase (6KSM).
