# Supplementary material for: Chemical Characterization, α-Glucosidase, α-Amylase and Lipase Inhibitory Properties of the Australian Honey Bee Propolis
Source: Foods. 2022 Jul 1;11(13):1964. doi: 10.3390/foods11131964 (PMC9266216; doi:10.3390/foods11131964)

Chemical characterisation, α-glucosidase, α-amylase and lipase inhibitory properties of the Australian honey bee propolis

Sabah Uddin[1], Peter R. Brooks[1,2], and Trong D. Tran[1,2,*]

[1] School of Science, Technology and Engineering, University of the Sunshine Coast, Maroochydore DC, Queensland 4558, Australia; ttran1@usc.edu.au

[2] Centre for Bioinnovation, University of the Sunshine Coast, Maroochydore DC, Queensland 4558, Australia.

* Correspondence: ttran1@usc.edu.au; Tel: +61 7 5459 4579

Supplementary Materials

Figure S1. ^{1}H Spectrum of **1** in MeOH-d_4

Figure S2. ^{13}C Spectrum of **1** in MeOH-d_4

Figure S3. HSQC Spectrum of **1** in MeOH-d_4

Figure S4. gCOSY Spectrum of **1** in MeOH-d_4

Figure S5. HMBC Spectrum of **1** in MeOH-d_4

Figure S6. ^{1}H Spectrum of **2** in MeOH-d_4

Figure S7. ^{13}C Spectrum of **2** in MeOH-d_4

Figure S8. HSQC Spectrum of **2** in MeOH-d_4

Figure S9. gCOSY Spectrum of **2** in MeOH-d_4

Figure S10. HMBC Spectrum of **2** in MeOH-d_4

Figure S11. ^{1}H Spectrum of **3** in MeOH-d_4

Figure S12. ^{13}C Spectrum of **3** in MeOH-d_4

Figure S13. HSQC Spectrum of **3** in MeOH-d_4

Figure S14. gCOSY Spectrum of **3** in MeOH-d_4

Figure S15. HMBC Spectrum of **3** in MeOH-d_4

Figure S16. ^{1}H Spectrum of **4** in MeOH-d_4

Figure S17. ^{13}C Spectrum of **4** in MeOH-d_4

Figure S18. HSQC Spectrum of **4** in MeOH-d_4

Figure S19. gCOSY Spectrum of **4** in MeOH-d_4

Figure S20. HMBC Spectrum of **4** in MeOH-d_4

Figure S21. ^{1}H Spectrum of **5** in $CHCl_3$-*d*

Figure S22. ^{13}C Spectrum of **5** in $CHCl_3$-*d*

Figure S23. HSQC Spectrum of **5** in $CHCl_3$-*d*

Figure S24. gCOSY Spectrum of **5** in $CHCl_3$-*d*

Figure S25. HMBC Spectrum of **5** in $CHCl_3$-*d*

Figure S26. ^{1}H Spectrum of **6** in MeOH-d_4

Figure S27. ^{13}C Spectrum of **6** in MeOH-d_4

Figure S28. HSQC Spectrum of **6** in MeOH-d_4

Figure S29. gCOSY Spectrum of **6** in MeOH-d_4

Figure S30. HMBC Spectrum of **6** in MeOH-d_4

Figure S31. Two-dimensional diagrams showing non-covalent interactions of **1**-**9** and acarbose (**10**) with α-glucosidase (2ZQ0)

Figure S32. Two-dimensional diagrams showing non-covalent interactions of **1**-**9** and acarbose (**10**) with α-amylase (2QV4)

Figure S33. Two-dimensional diagrams showing non-covalent interactions of **1**-**9** and orlistat (**11**) with lipase (6KSM)

Figure S1. ^{1}H Spectrum of **1** in MeOH-*d*$_4$.

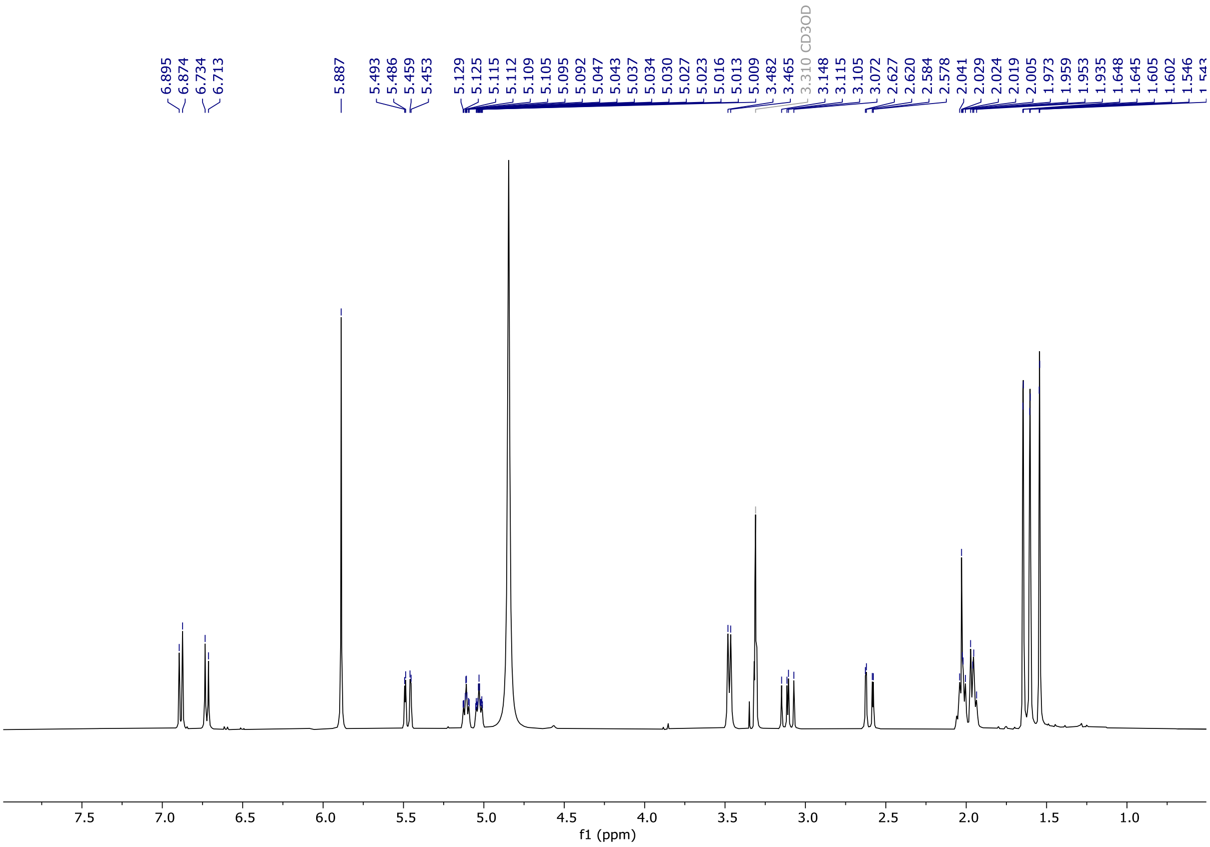

Figure S2. ^{13}C Spectrum of **1** in MeOH-*d*$_4$.

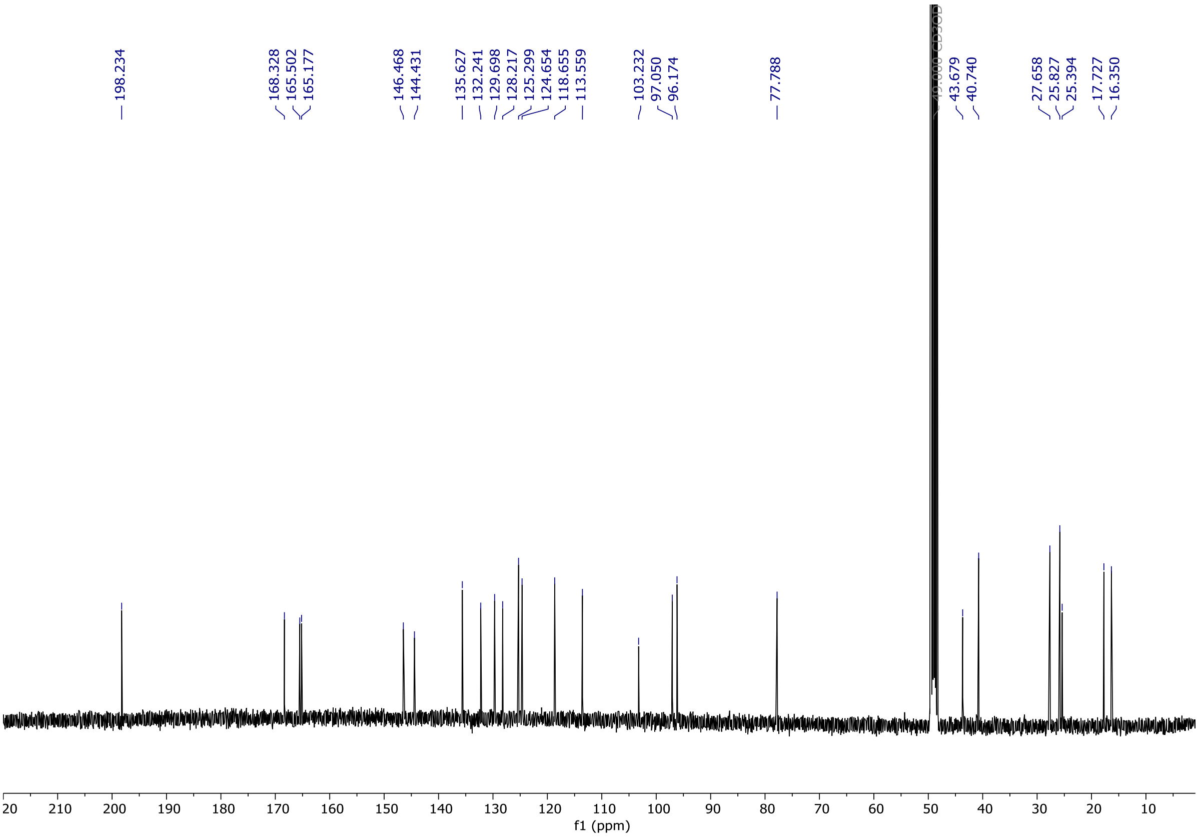

Figure S3. HSQC Spectrum of **1** in MeOH-d_4.

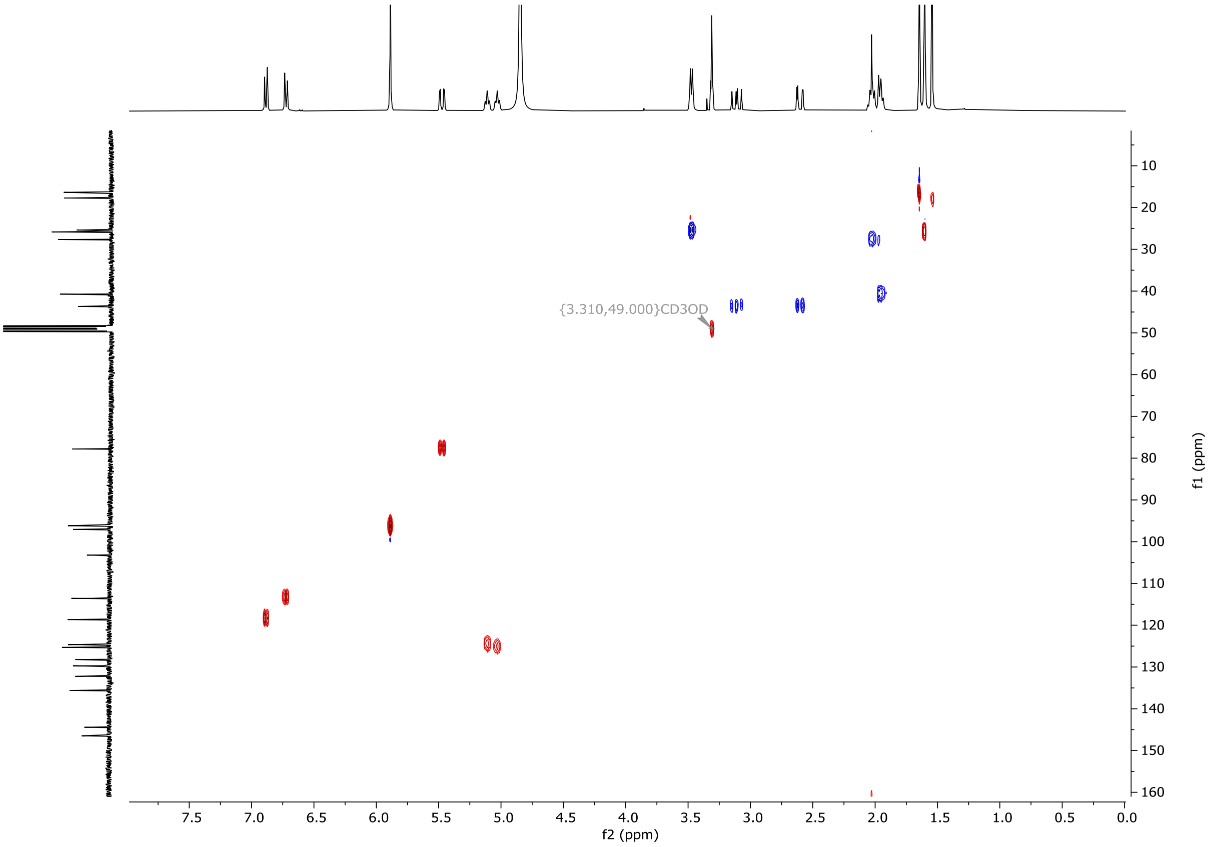

Figure S4. **g**COSY Spectrum of **1** in MeOH-d_4.

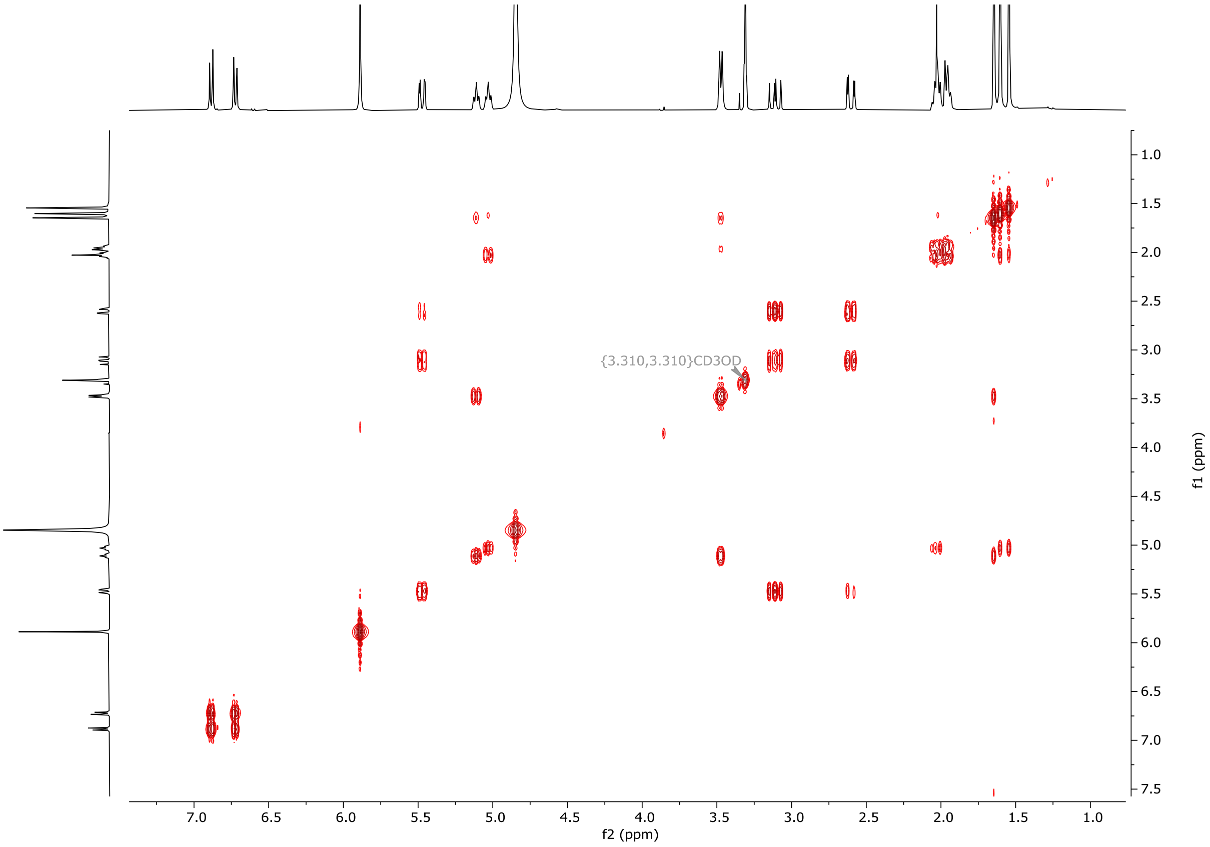

Figure S5. HMBC Spectrum of **1** in MeOH-*d*$_4$.

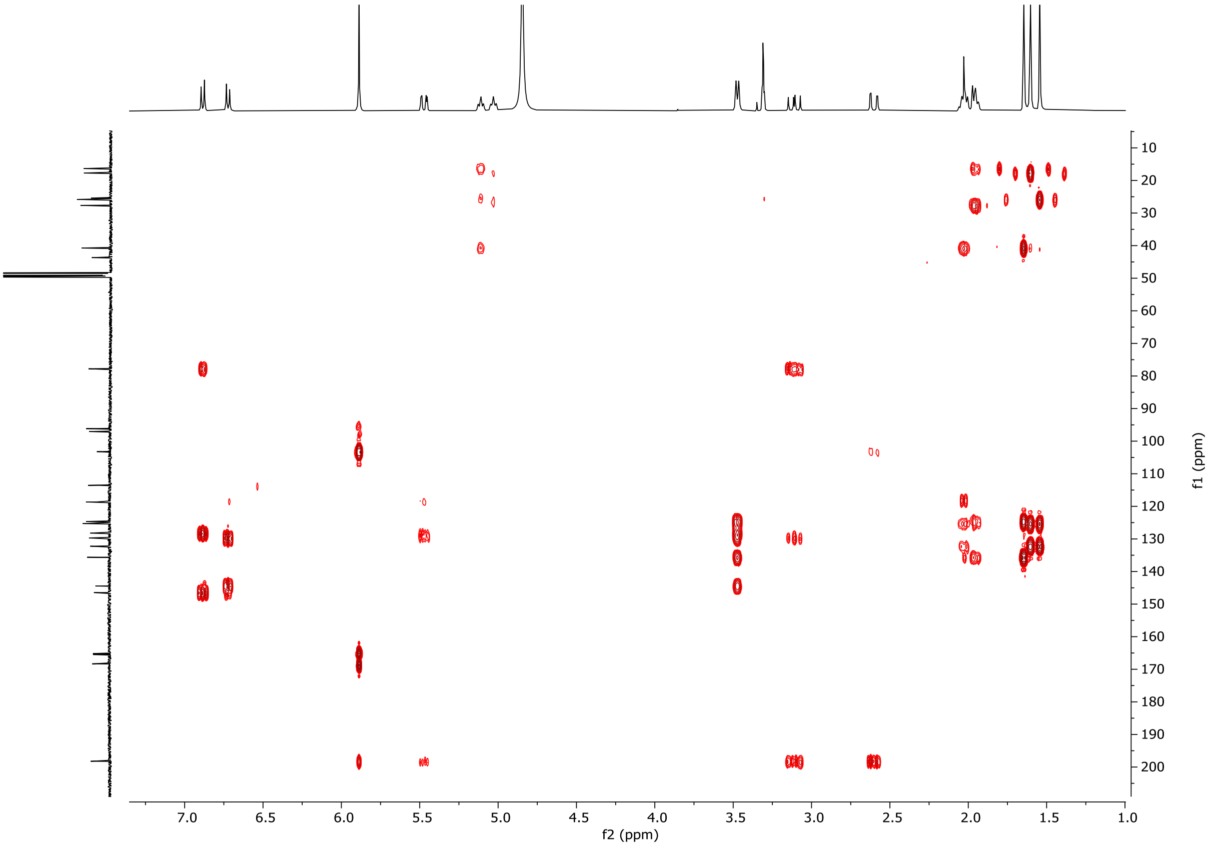

Figure S6. ^{1}H Spectrum of **2** in MeOH-*d*$_4$.

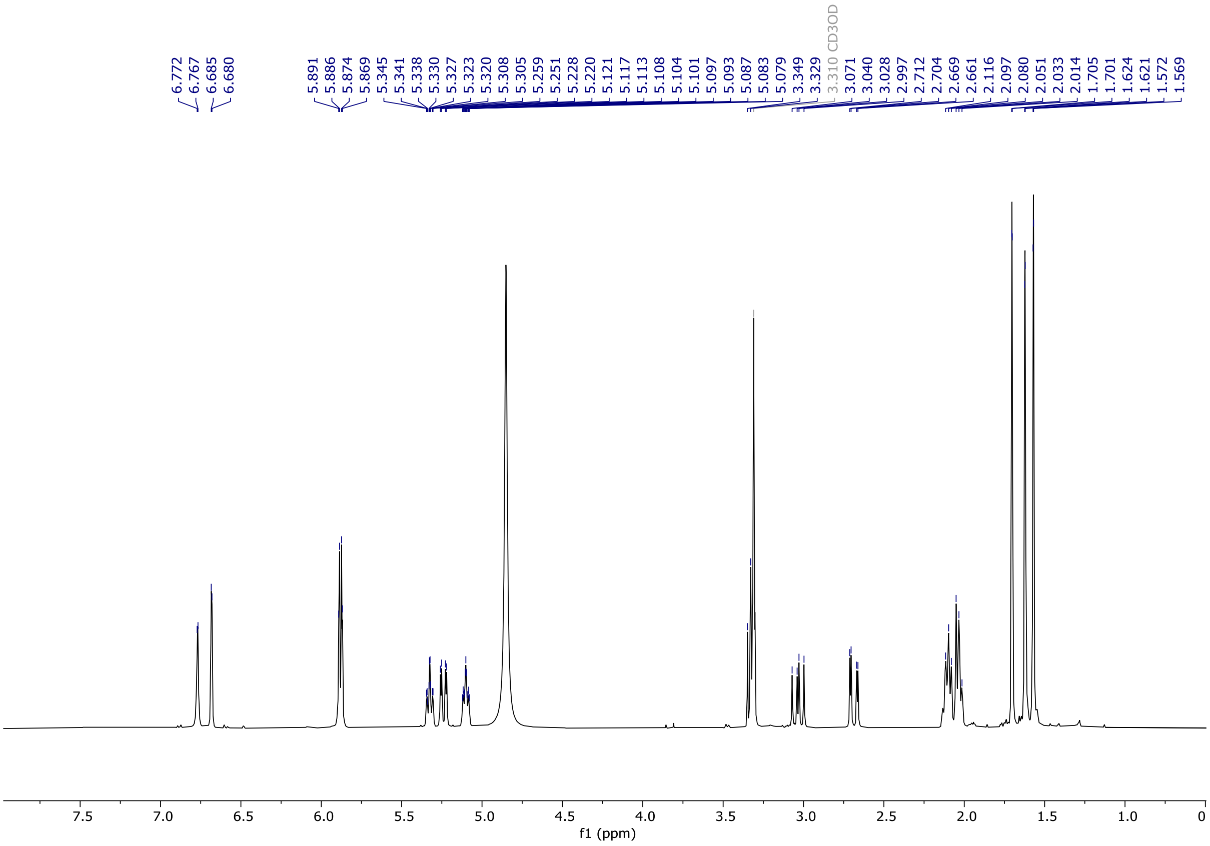

Figure S7. ^{13}C Spectrum of **2** in MeOH-d_4.

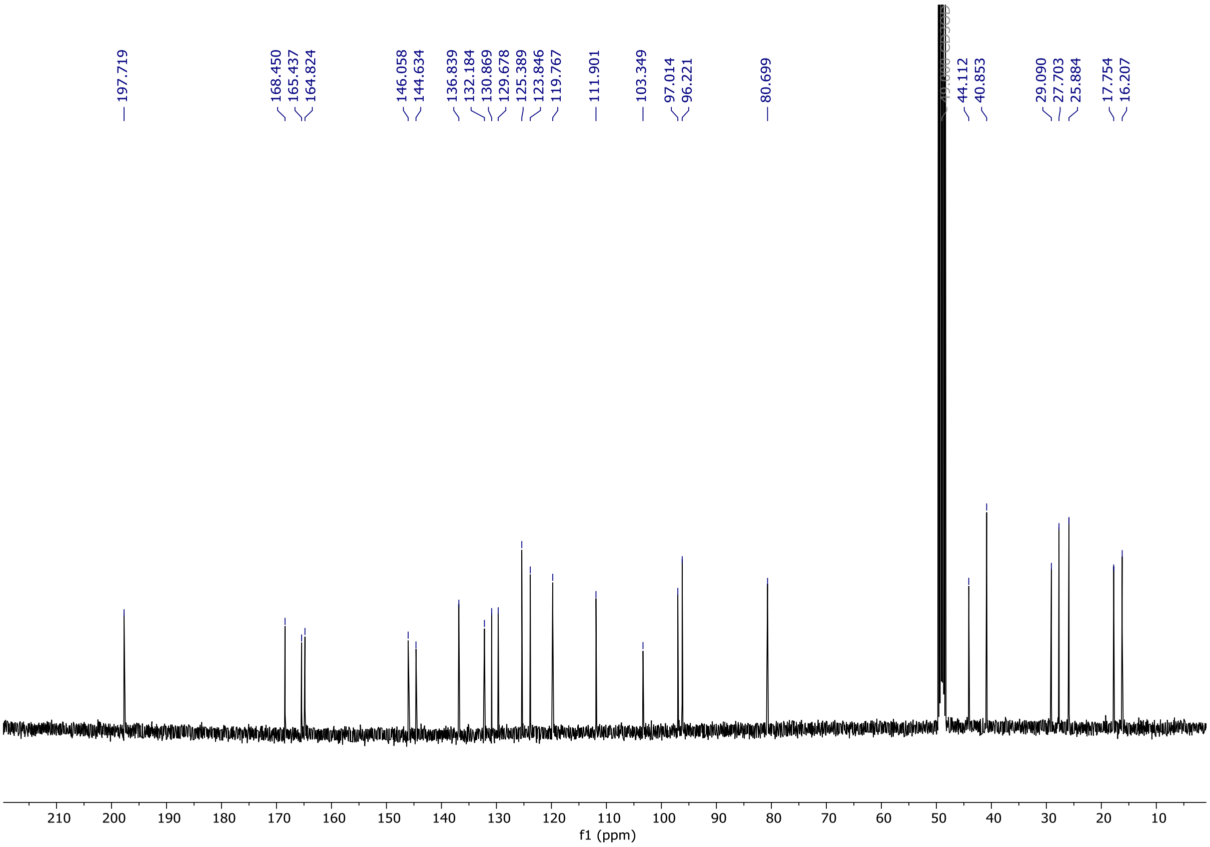

Figure S8. HSQC Spectrum of **2** in MeOH-d_4.

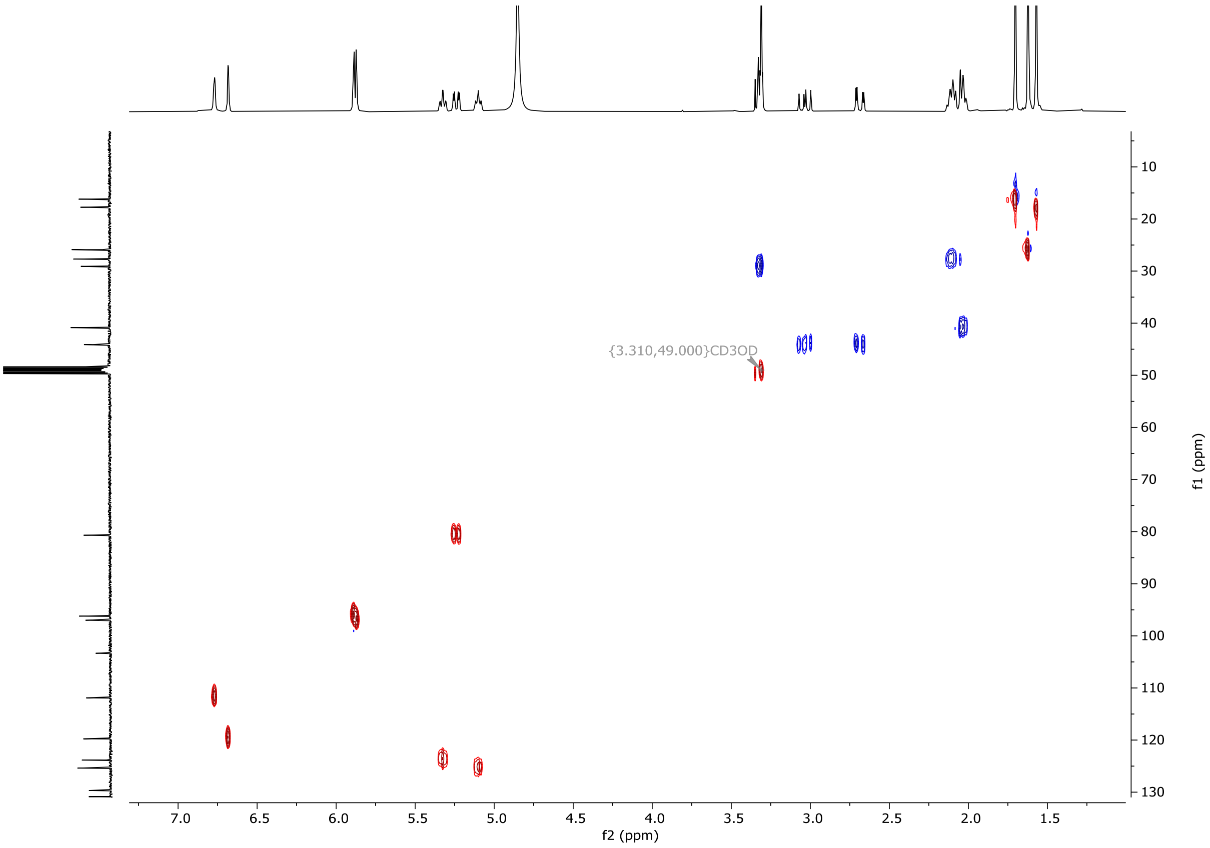

Figure S9. **g**COSY Spectrum of **2** in MeOH-*d*4.

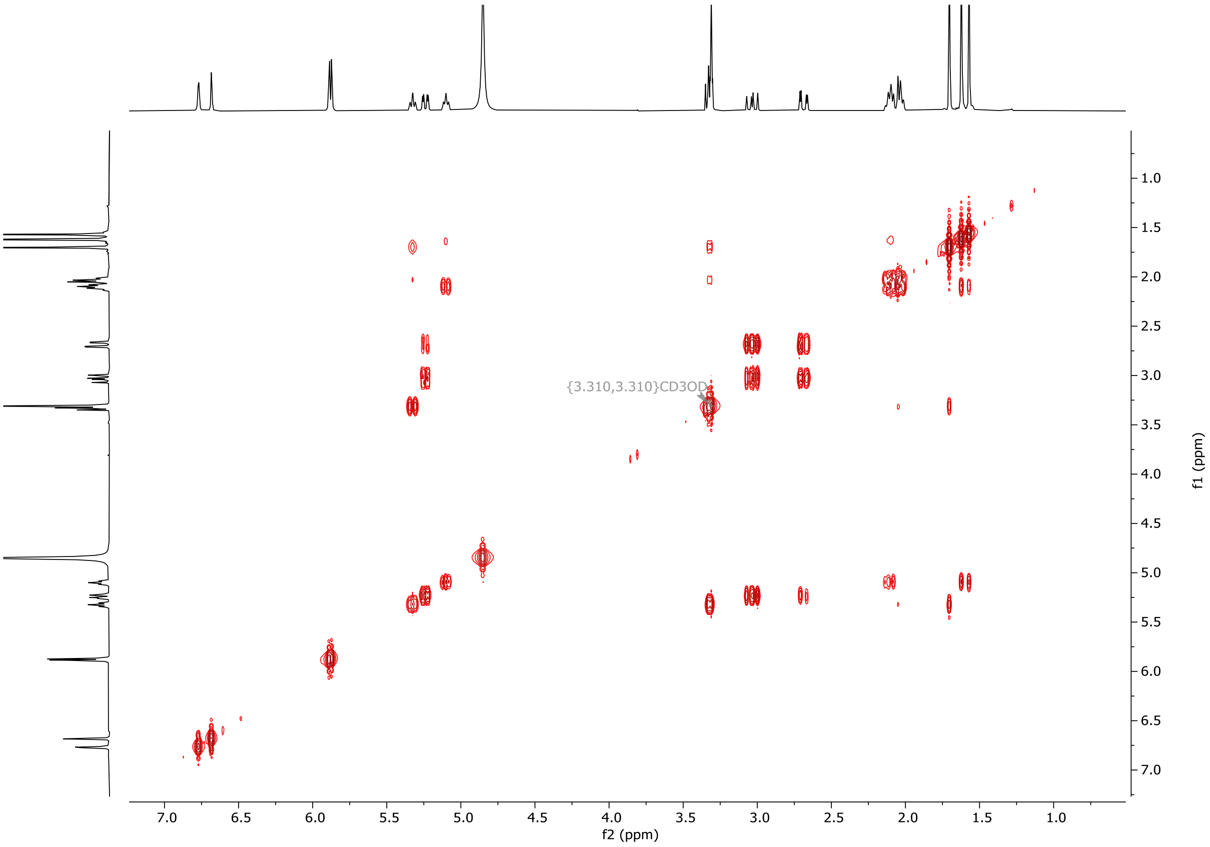

Figure S10. HMBC Spectrum of **2** in MeOH-*d*4.

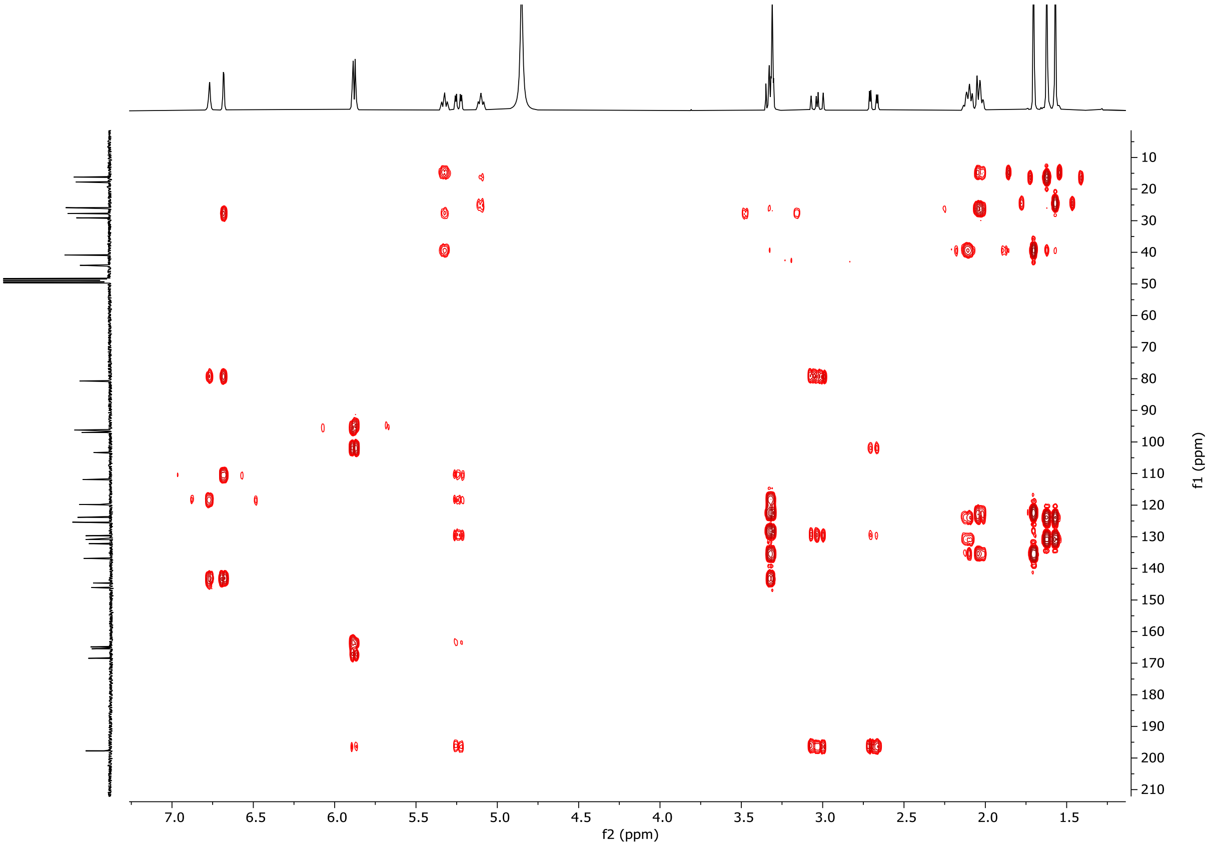

Figure S11. ^{1}H Spectrum of **3** in MeOH-d_4.

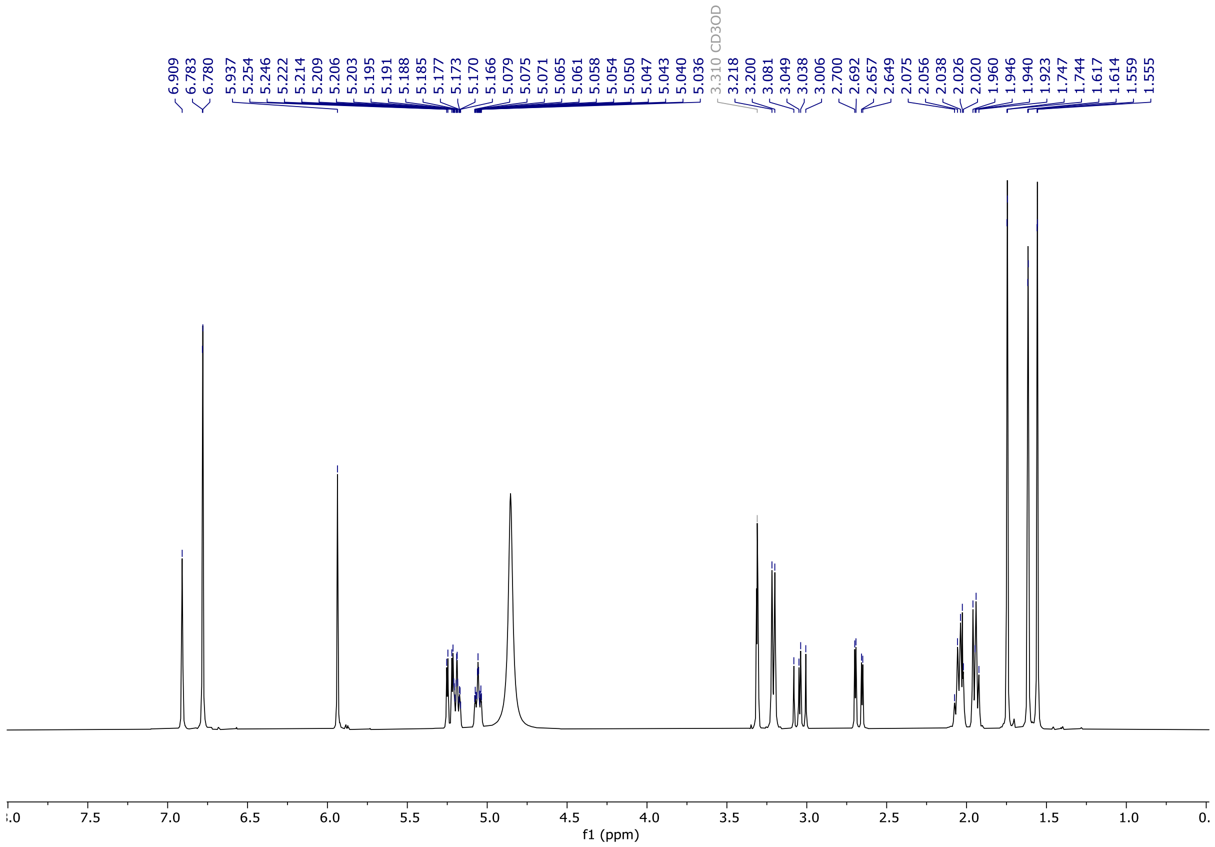

Figure S12. ^{13}C Spectrum of **3** in MeOH-d_4.

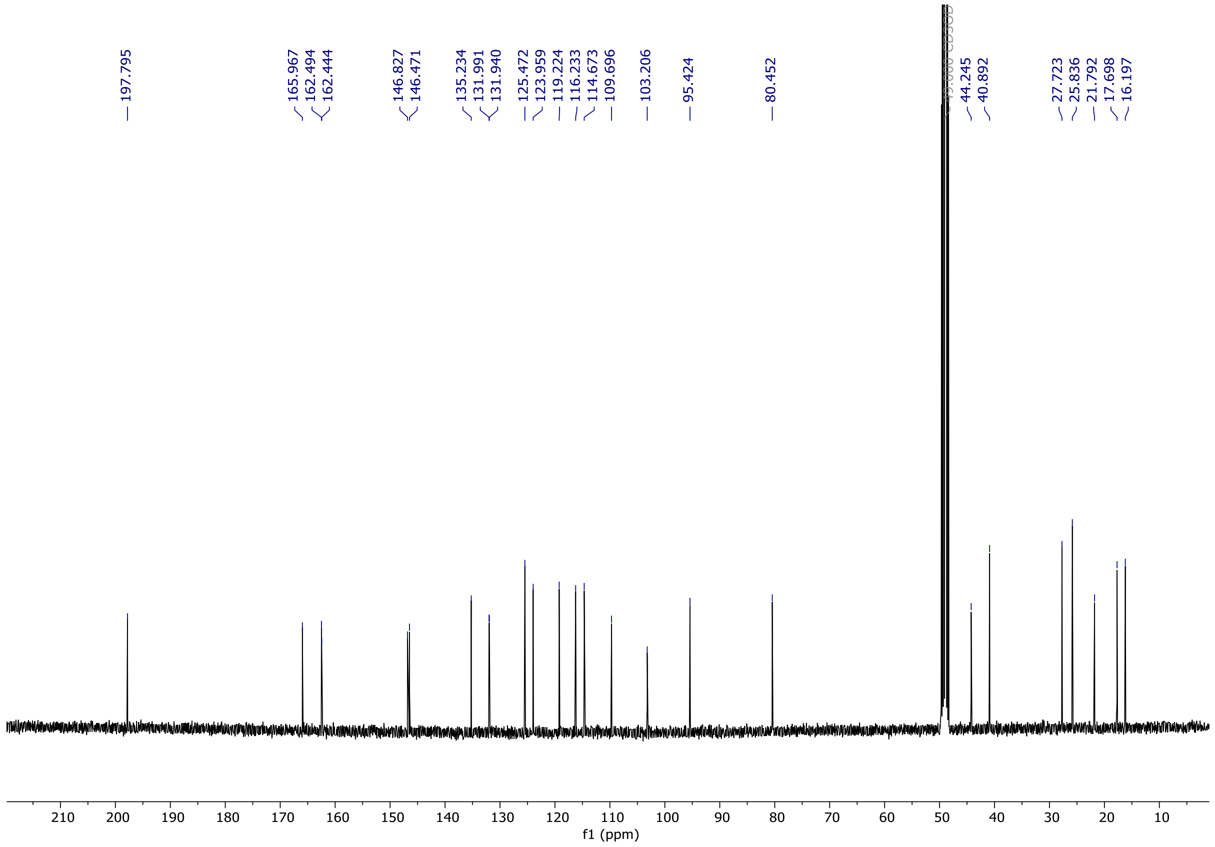

Figure S13. HSQC Spectrum of **3** in MeOH-d_4.

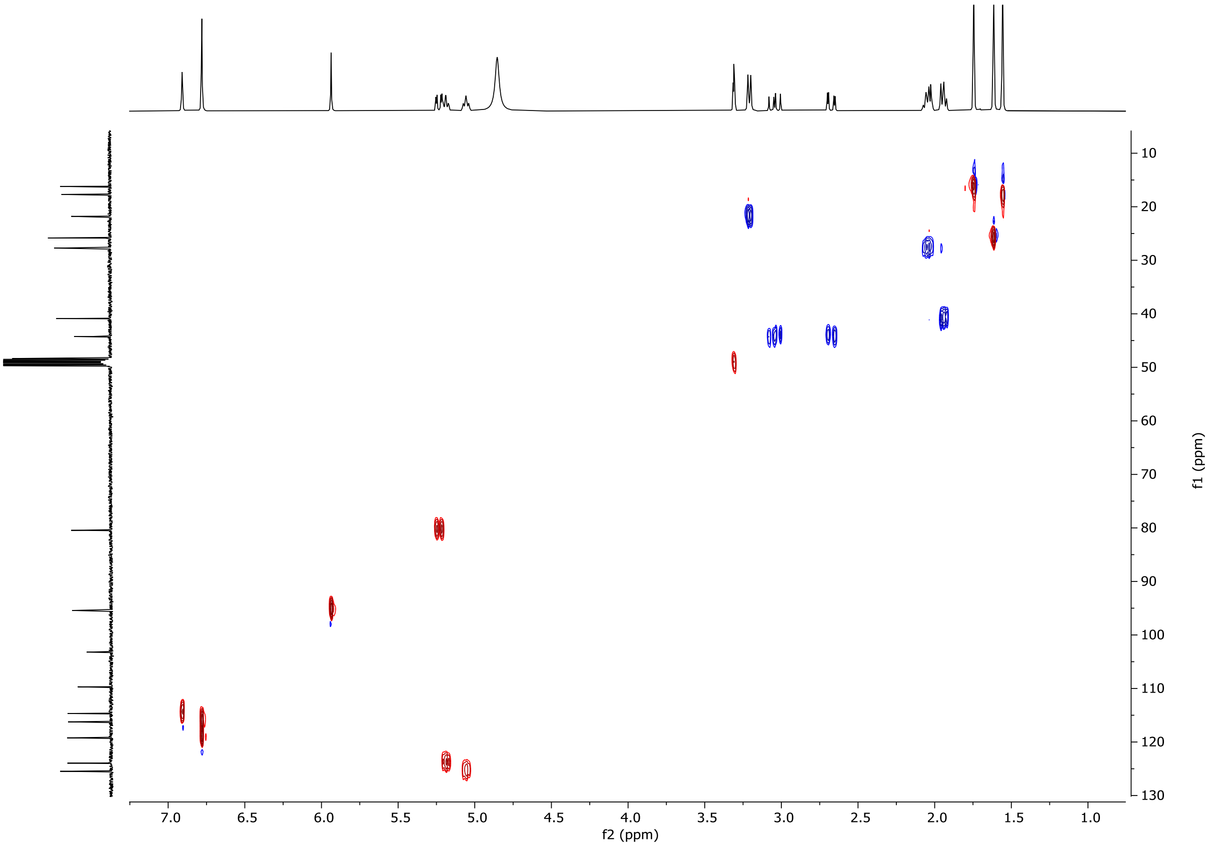

Figure S14. **g**COSY Spectrum of **3** in MeOH-d_4.

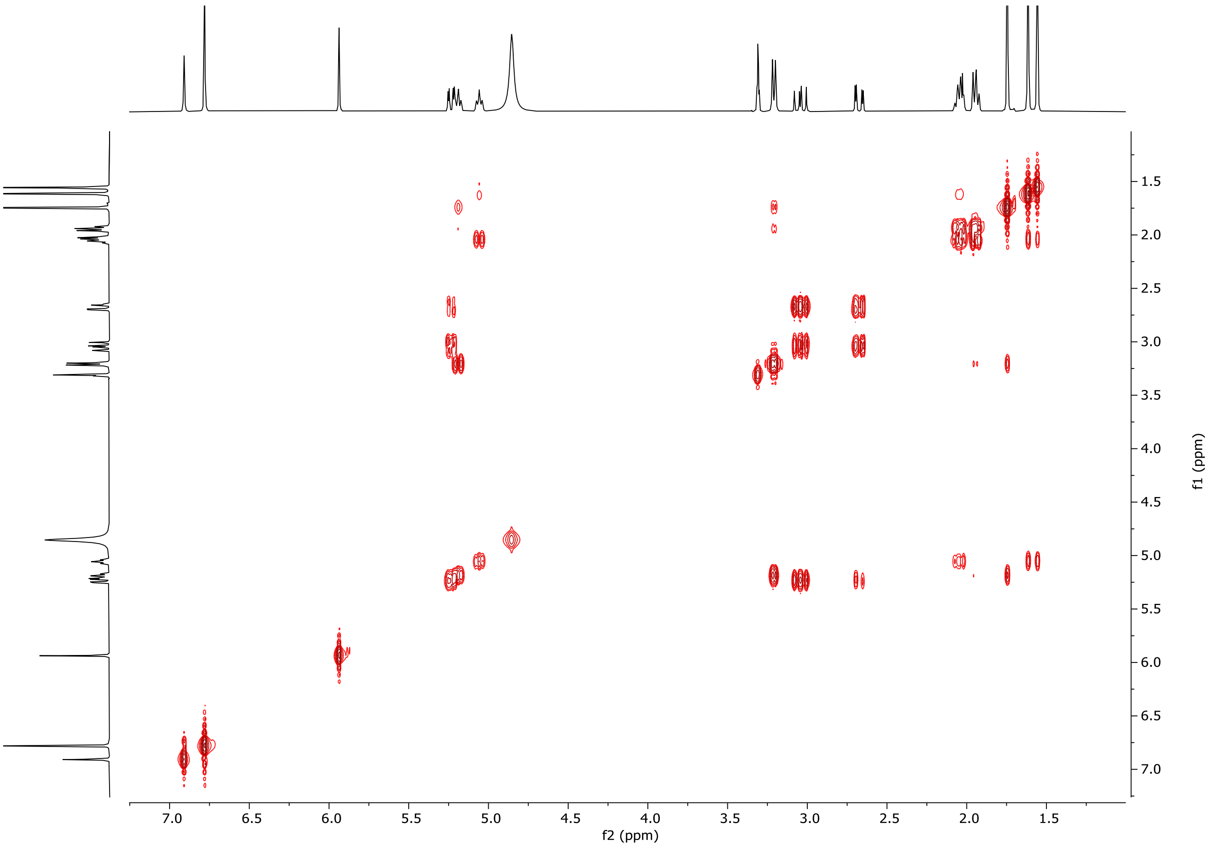

Figure S15. HMBC Spectrum of **3** in MeOH-d_4.

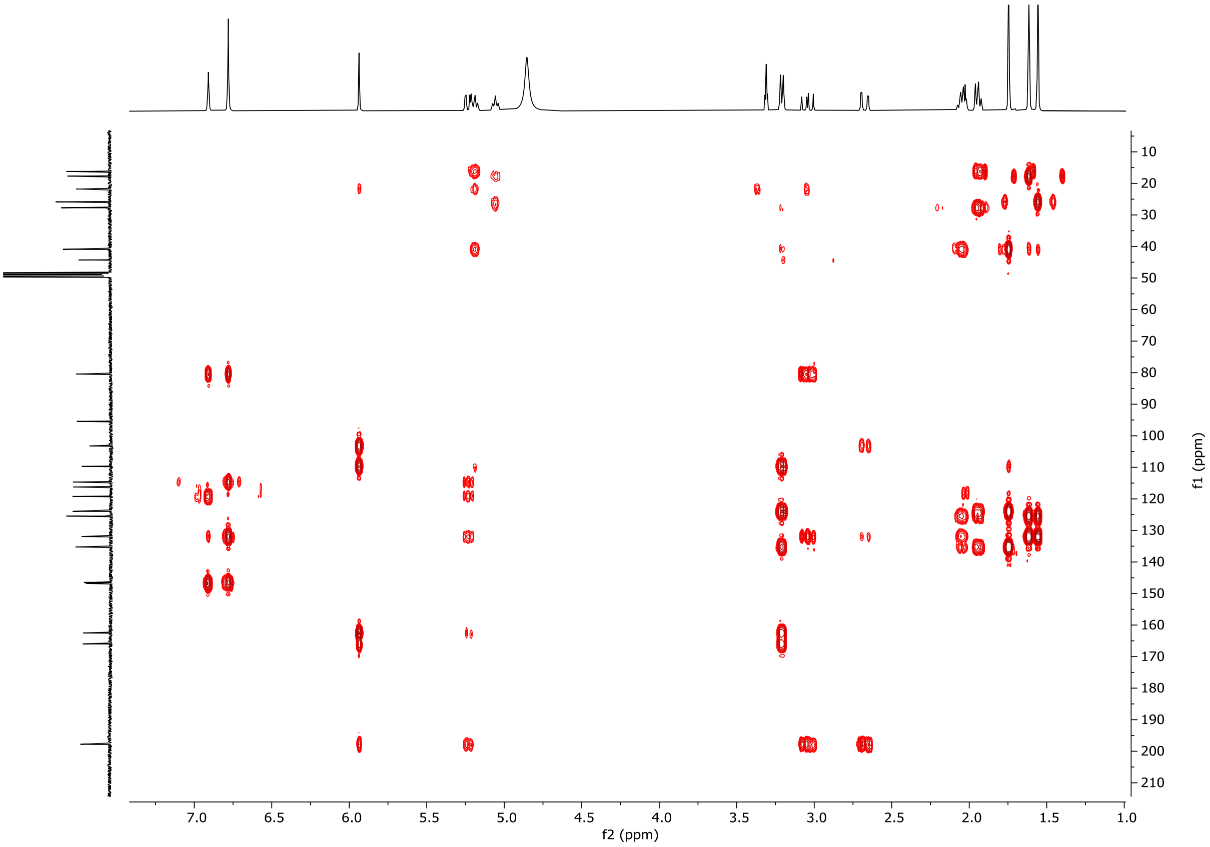

Figure S16. [1]H Spectrum of **4** in MeOH-d_4.

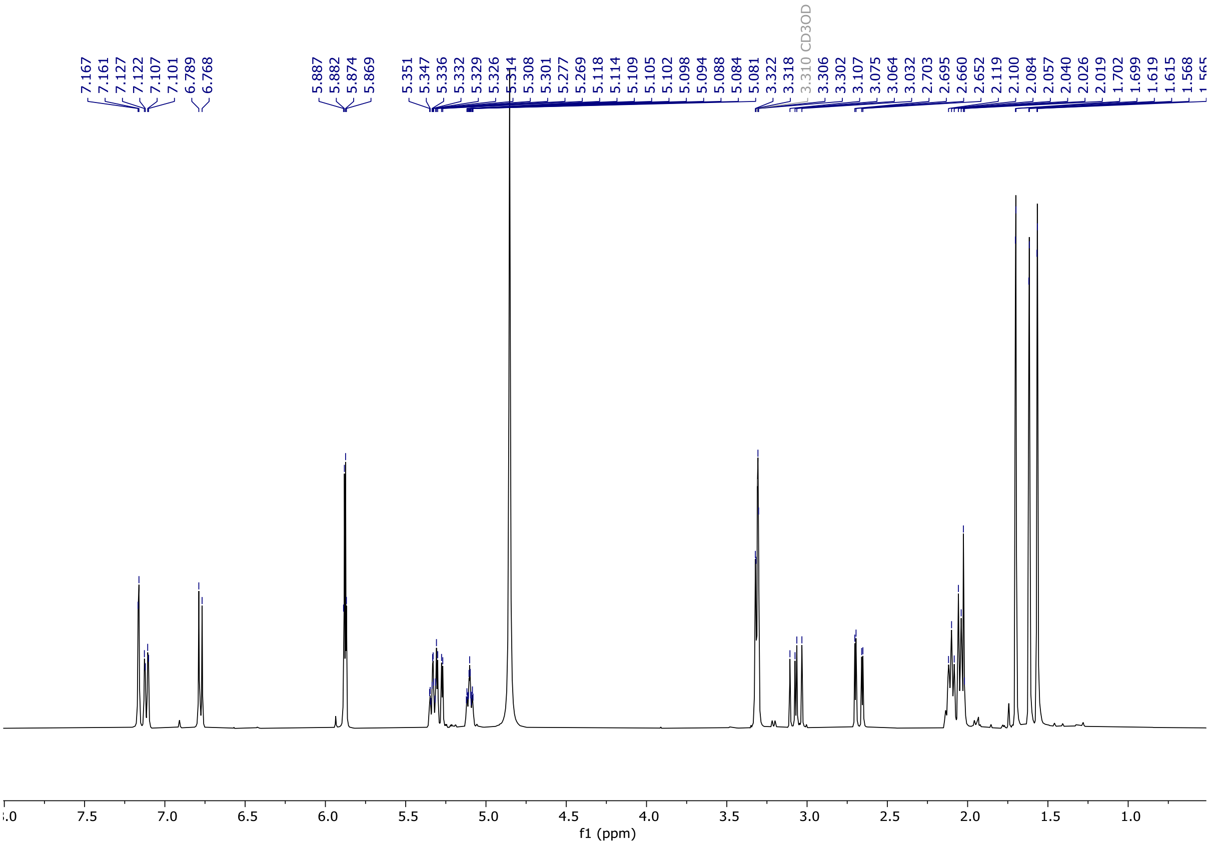

Figure S17. ^{13}C Spectrum of **4** in MeOH-d_4.

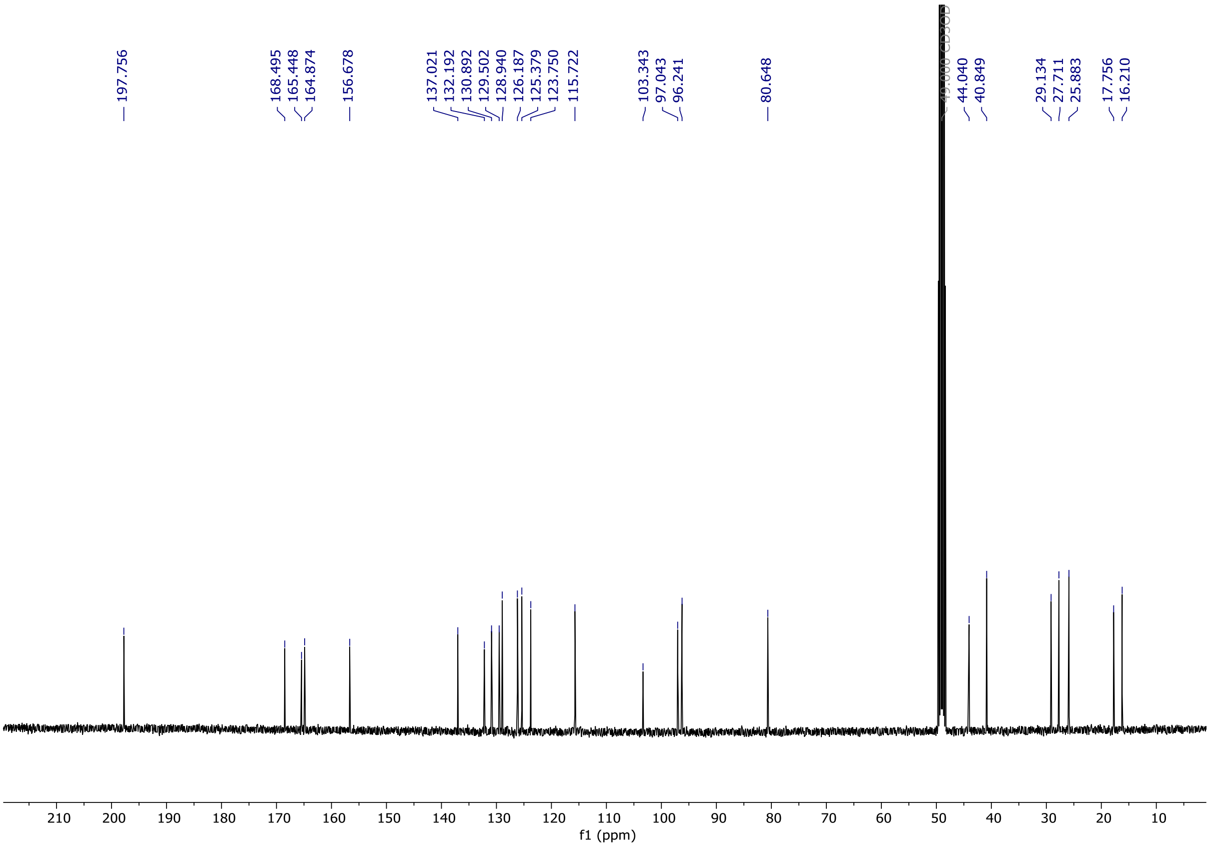

Figure S18. HSQC Spectrum of **4** in MeOH-d_4.

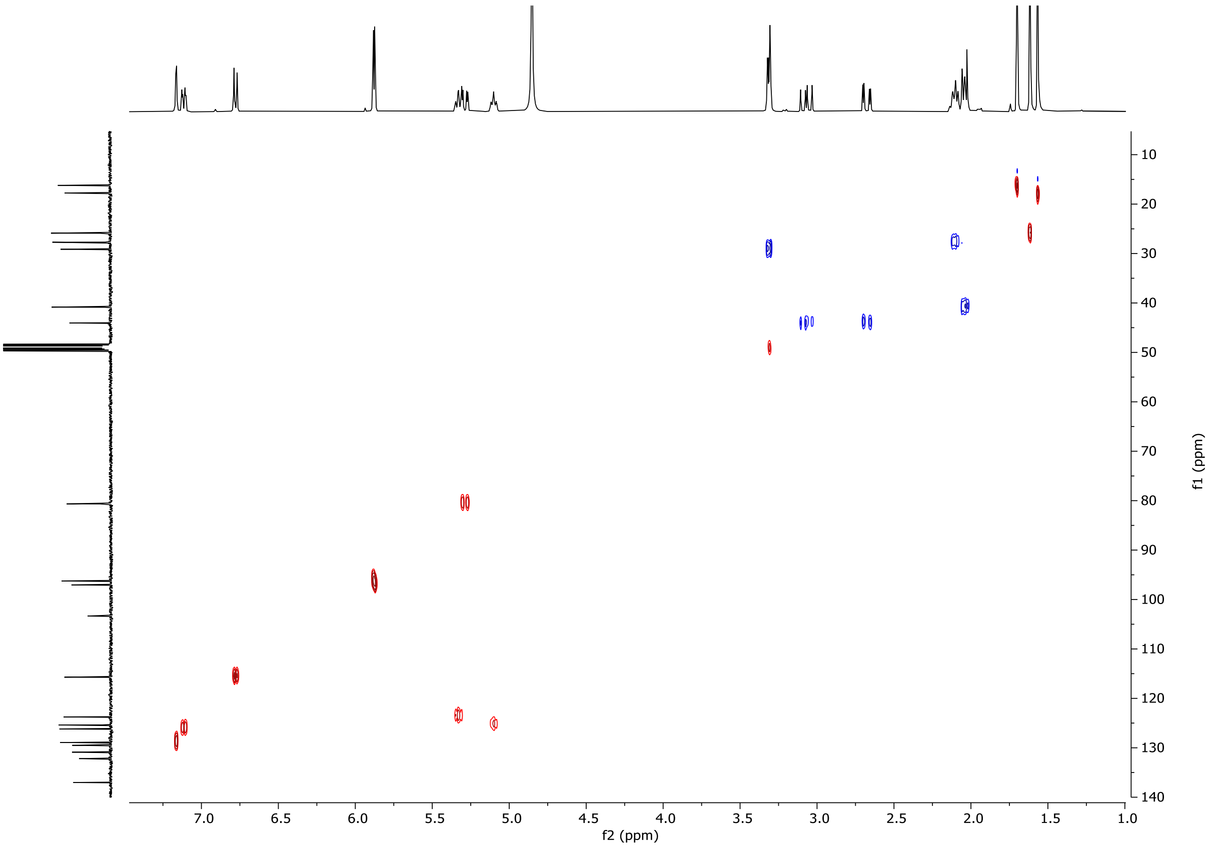

Figure S19. **g**COSY Spectrum of **4** in MeOH-*d*4.

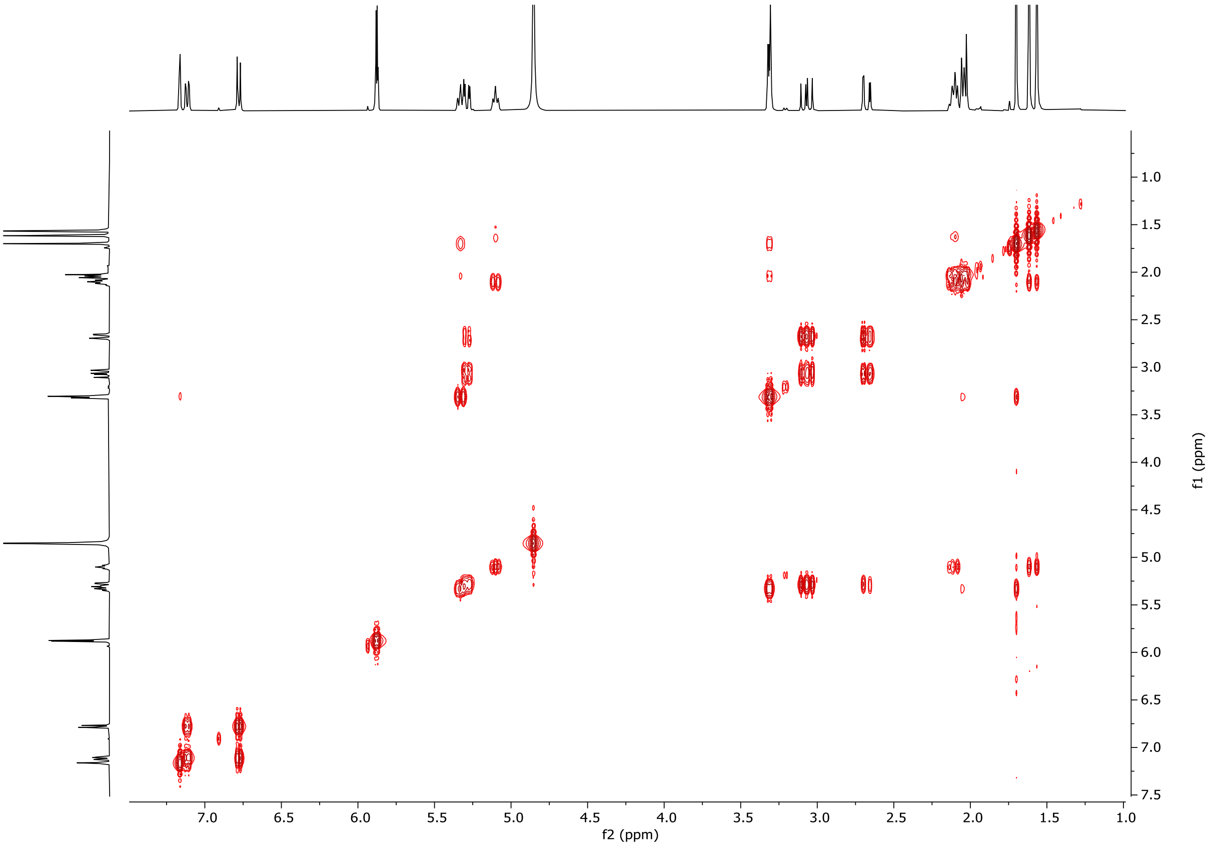

Figure S20. HMBC Spectrum of **4** in MeOH-*d*4.

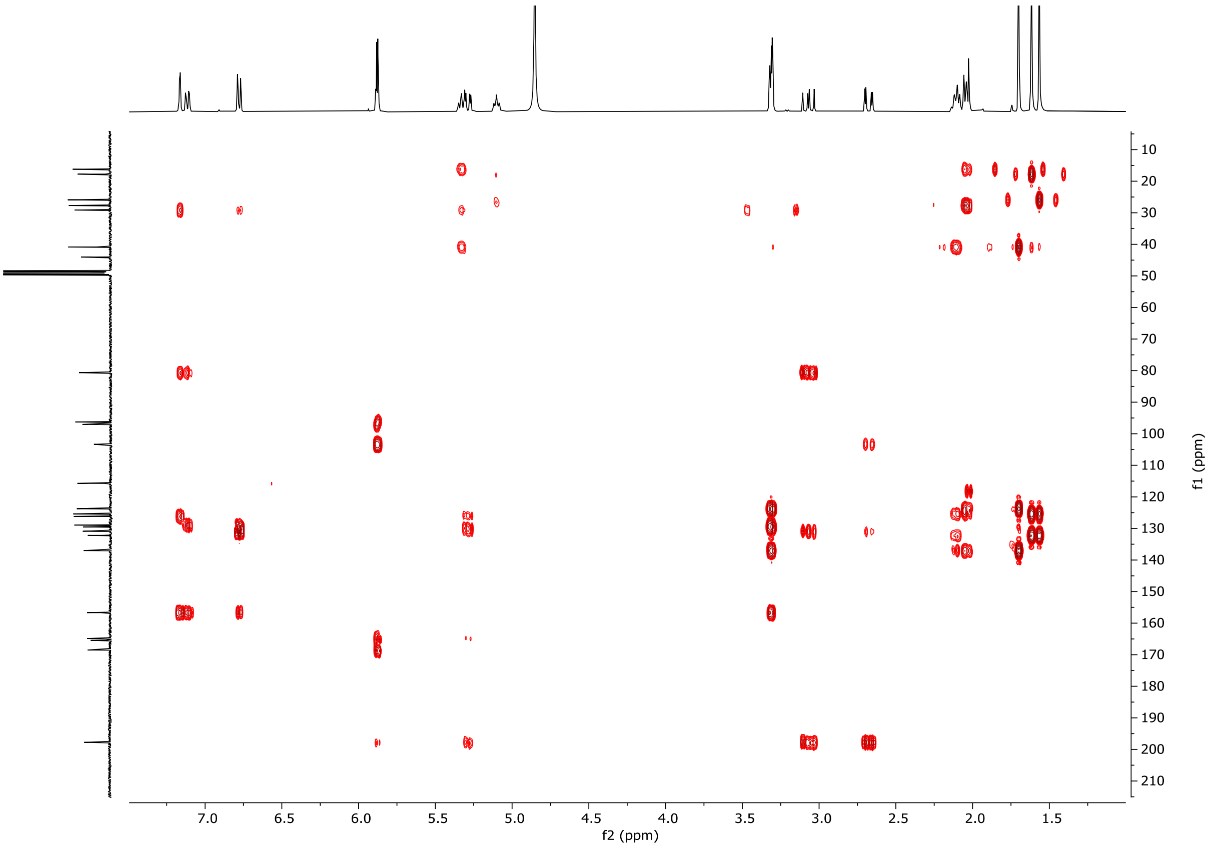

Figure S21. ^{1}H Spectrum of **5** in $CHCl_3$-*d*.

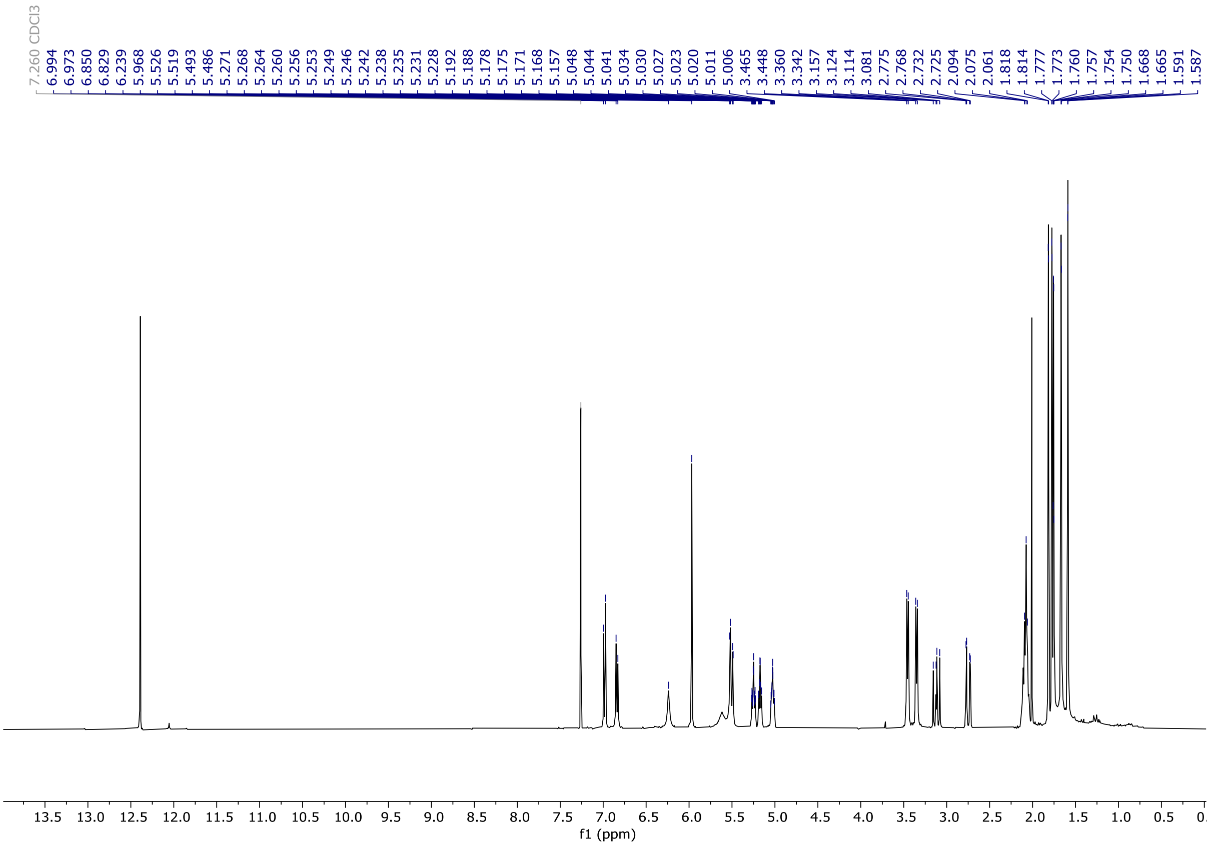

Figure S22. ^{13}C Spectrum of **5** in $CHCl_3$-*d*.

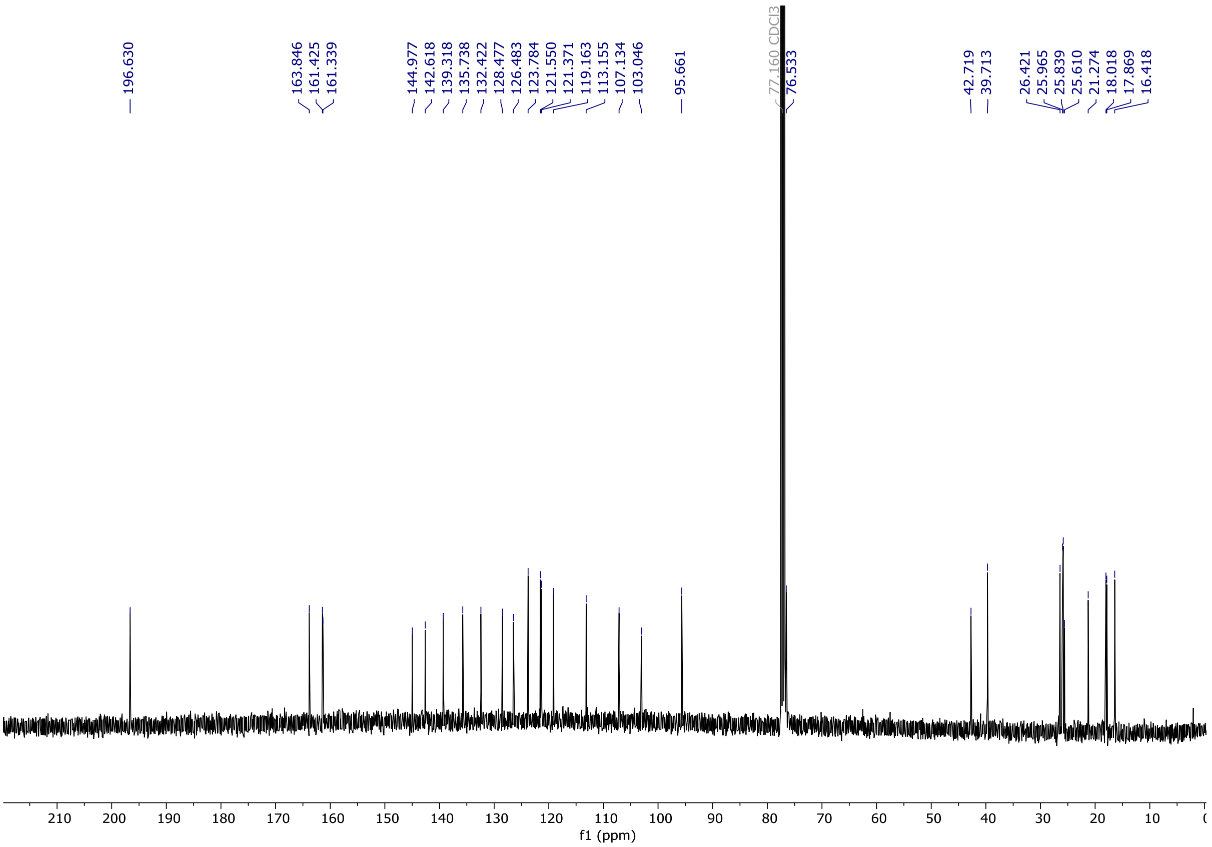

Figure S23. HSQC Spectrum of **5** in $CHCl_3$-*d*.

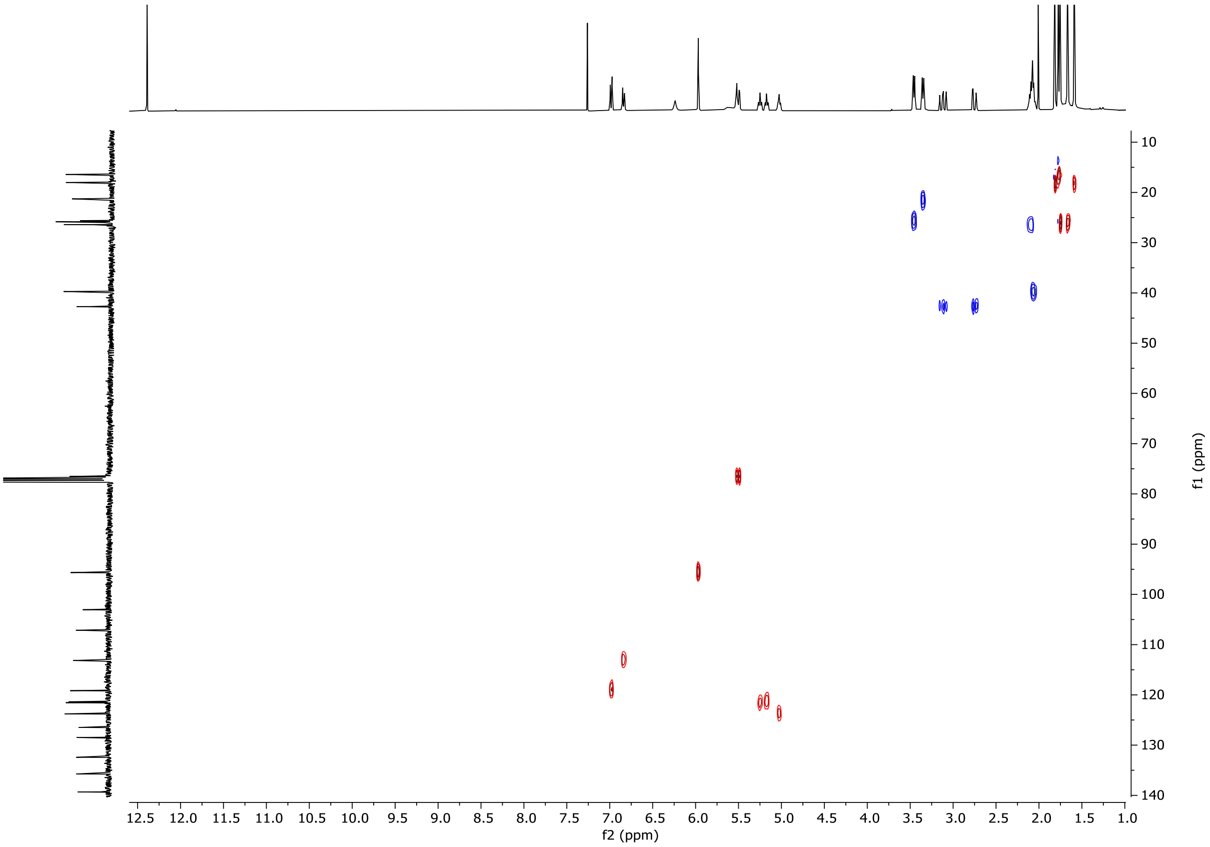

Figure S24. **g**COSY Spectrum of **5** in $CHCl_3$-*d*.

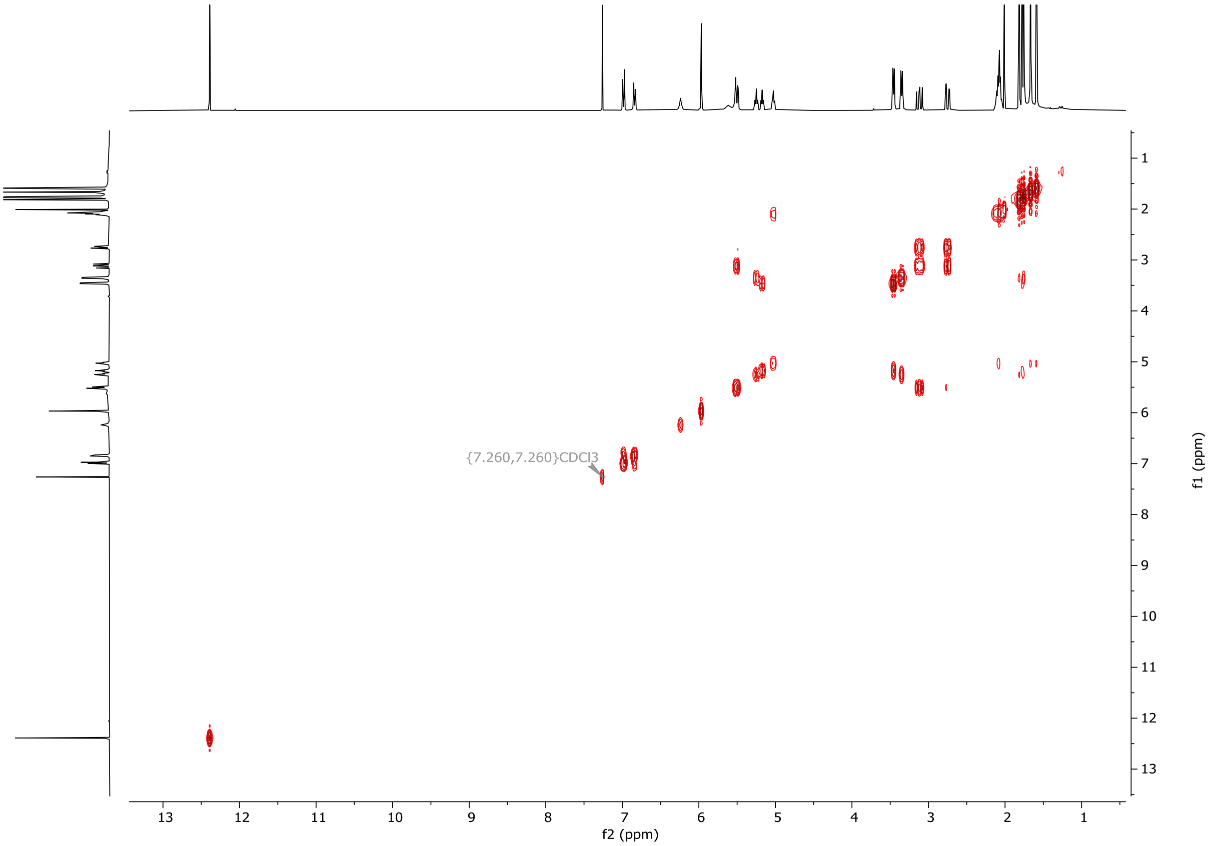

Figure S25. HMBC Spectrum of **5** in $CHCl_3$-*d*.

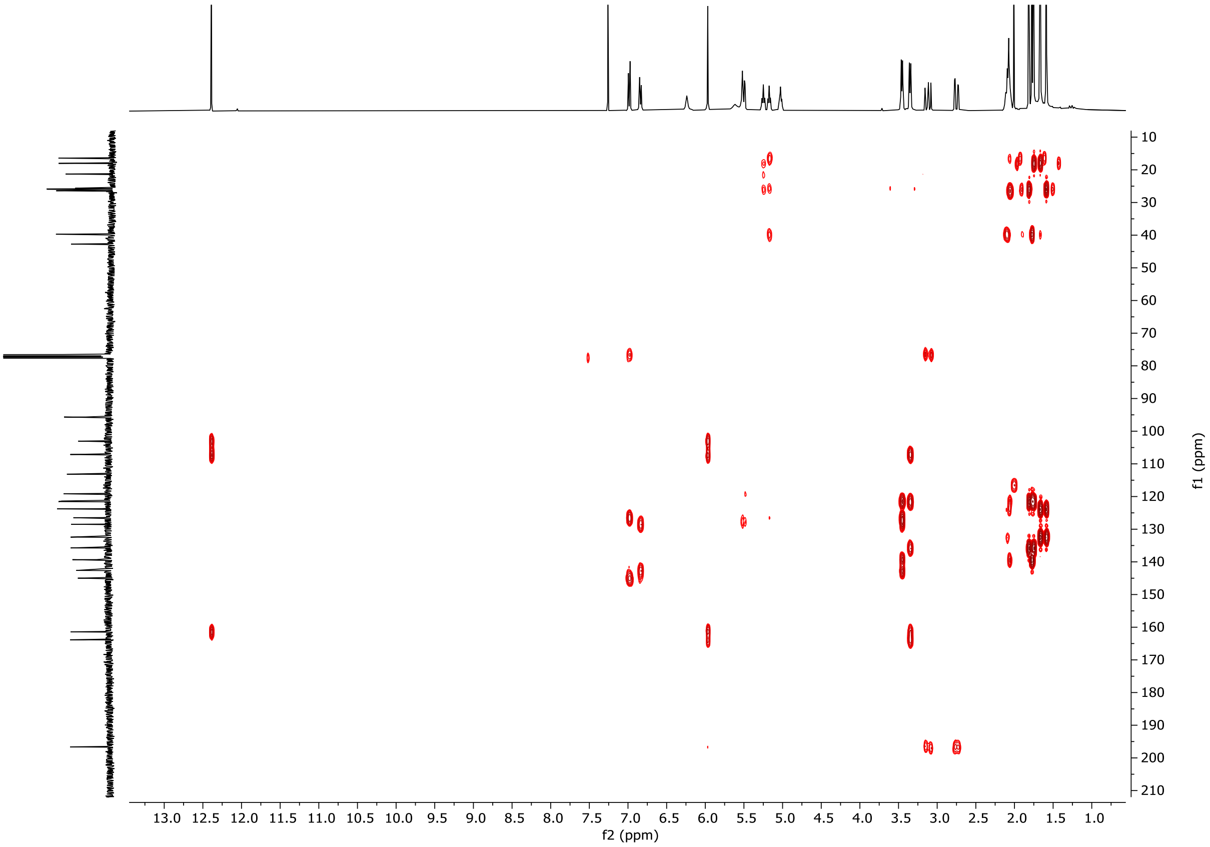

Figure S26. [1]H Spectrum of **6** in MeOH-*d*4.

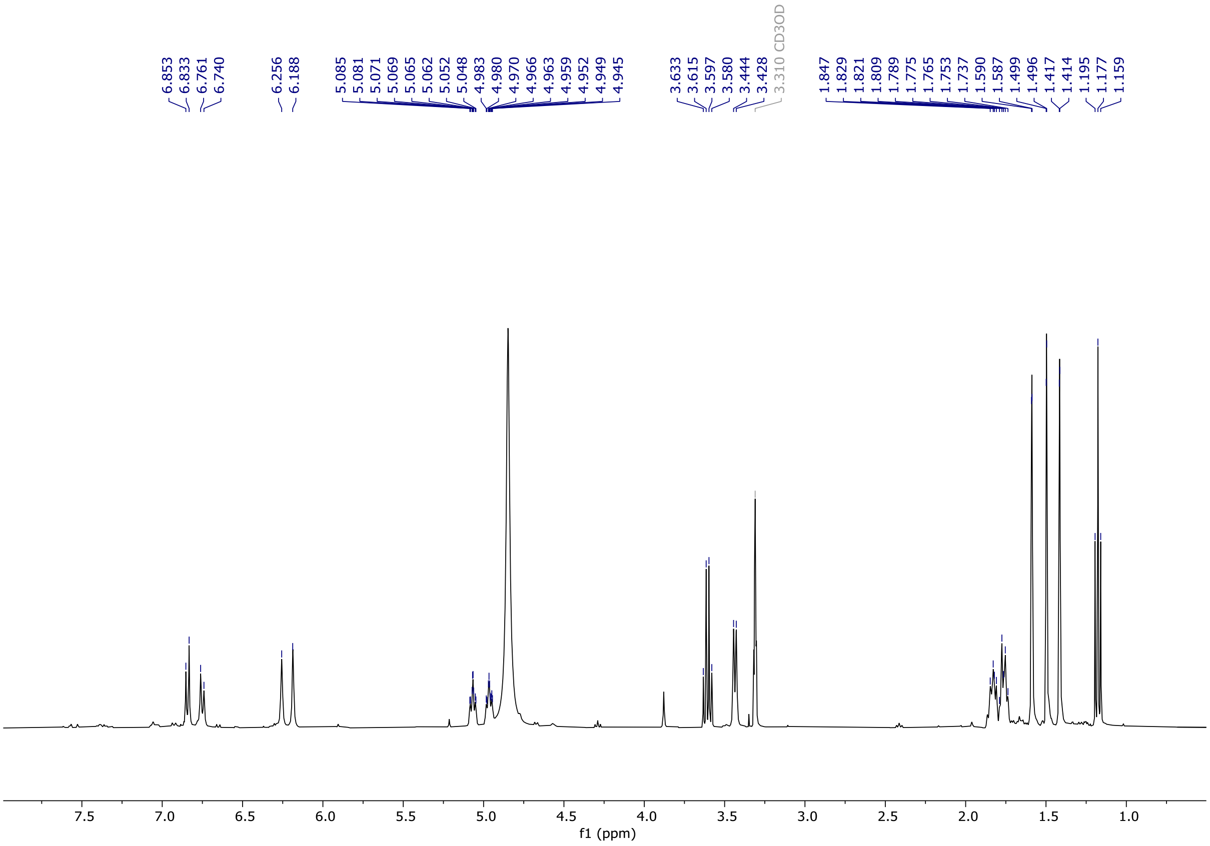

Figure S27. ^{13}C Spectrum of **6** in MeOH-*d*$_4$.

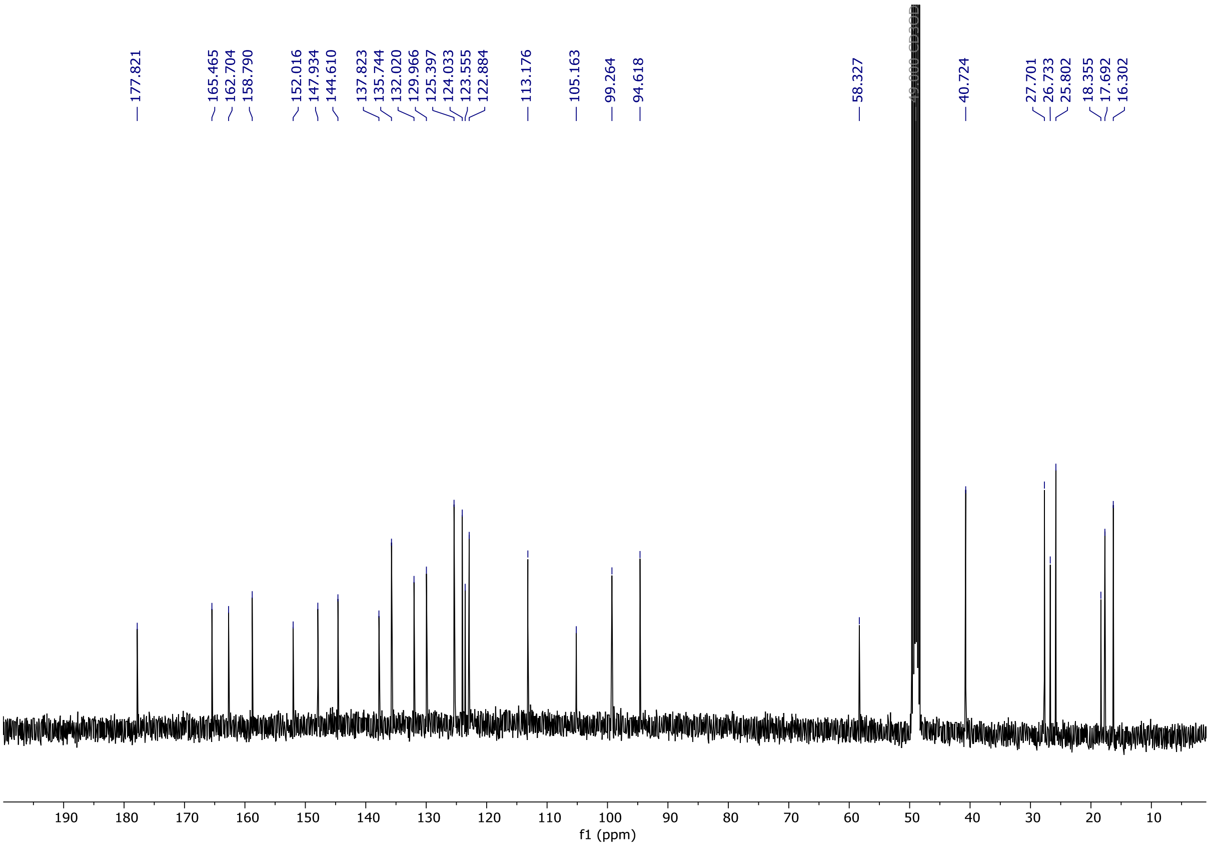

Figure S28. HSQC Spectrum of **6** in MeOH-*d*$_4$.

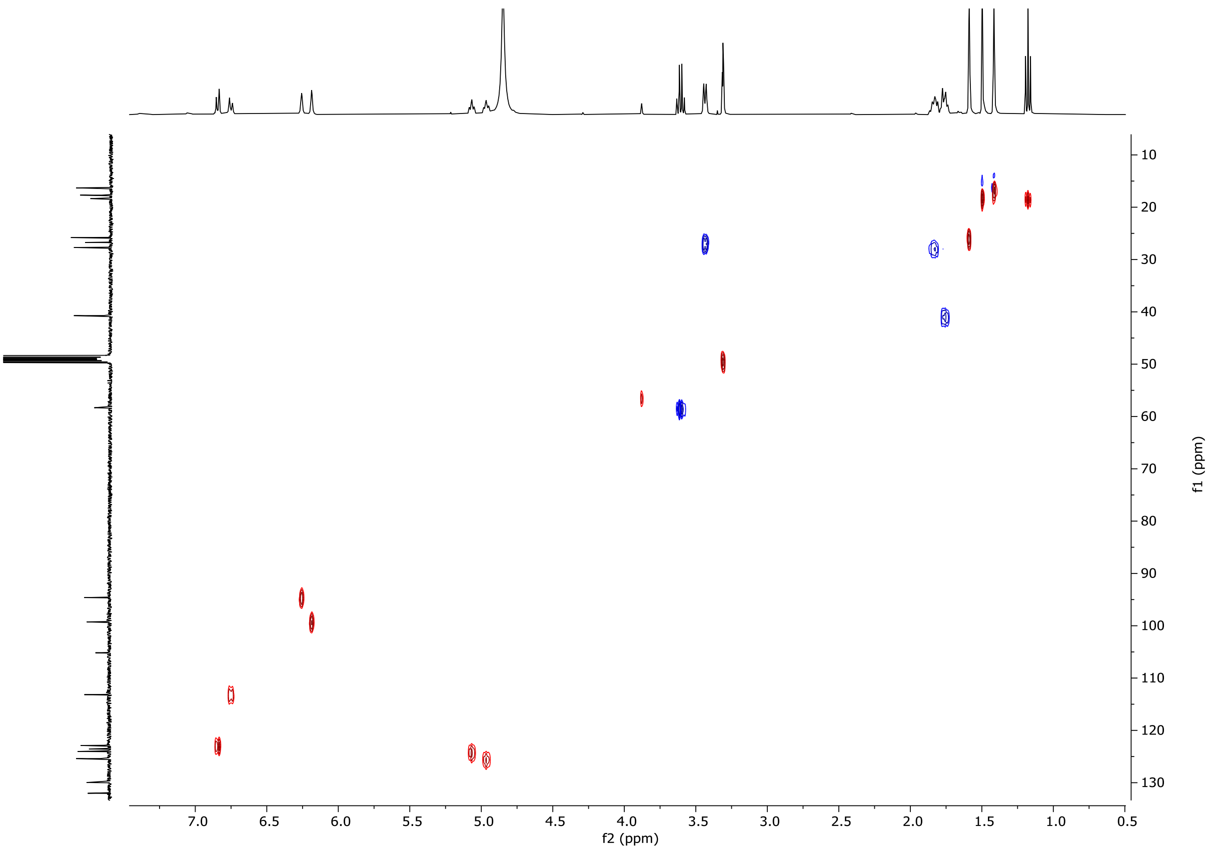

Figure S29. **g**COSY Spectrum of **6** in MeOH-*d*$_4$.

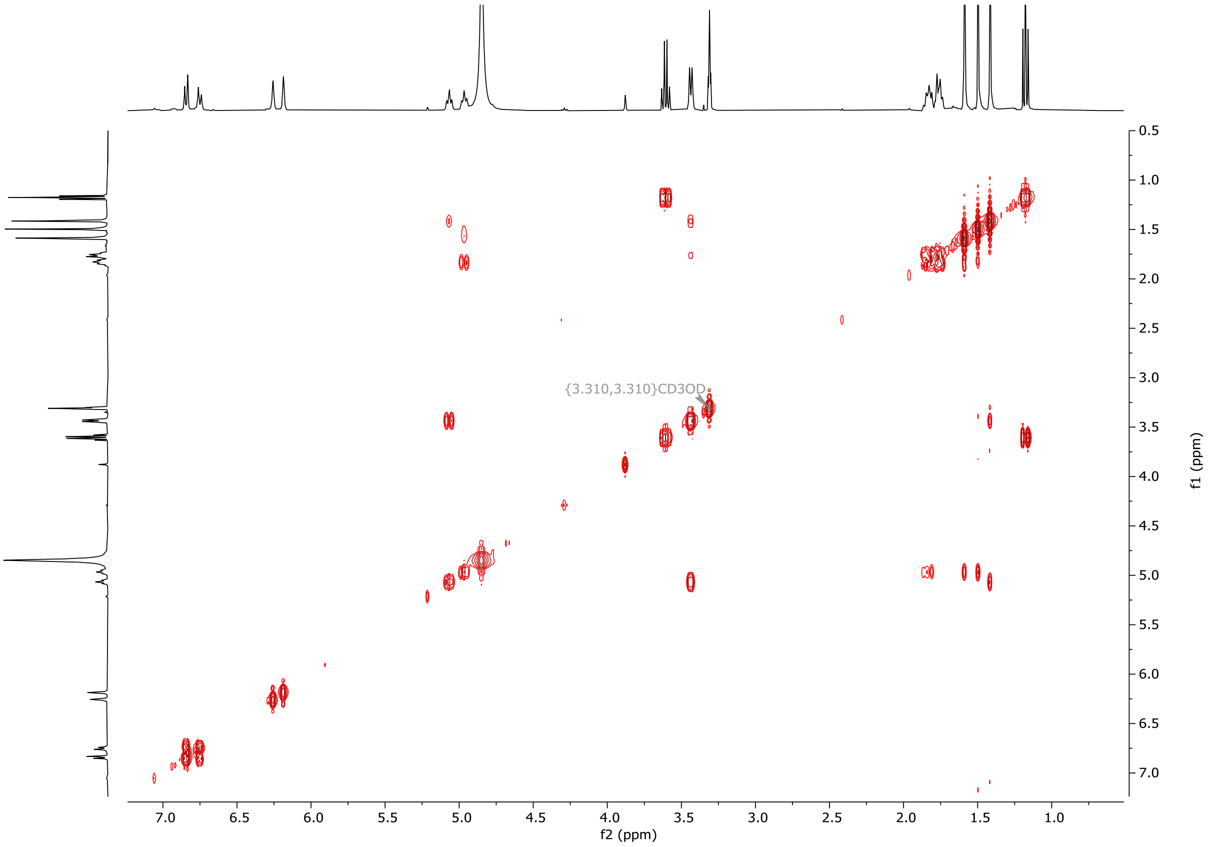

Figure S30. HMBC Spectrum of **6** in MeOH-*d*$_4$.

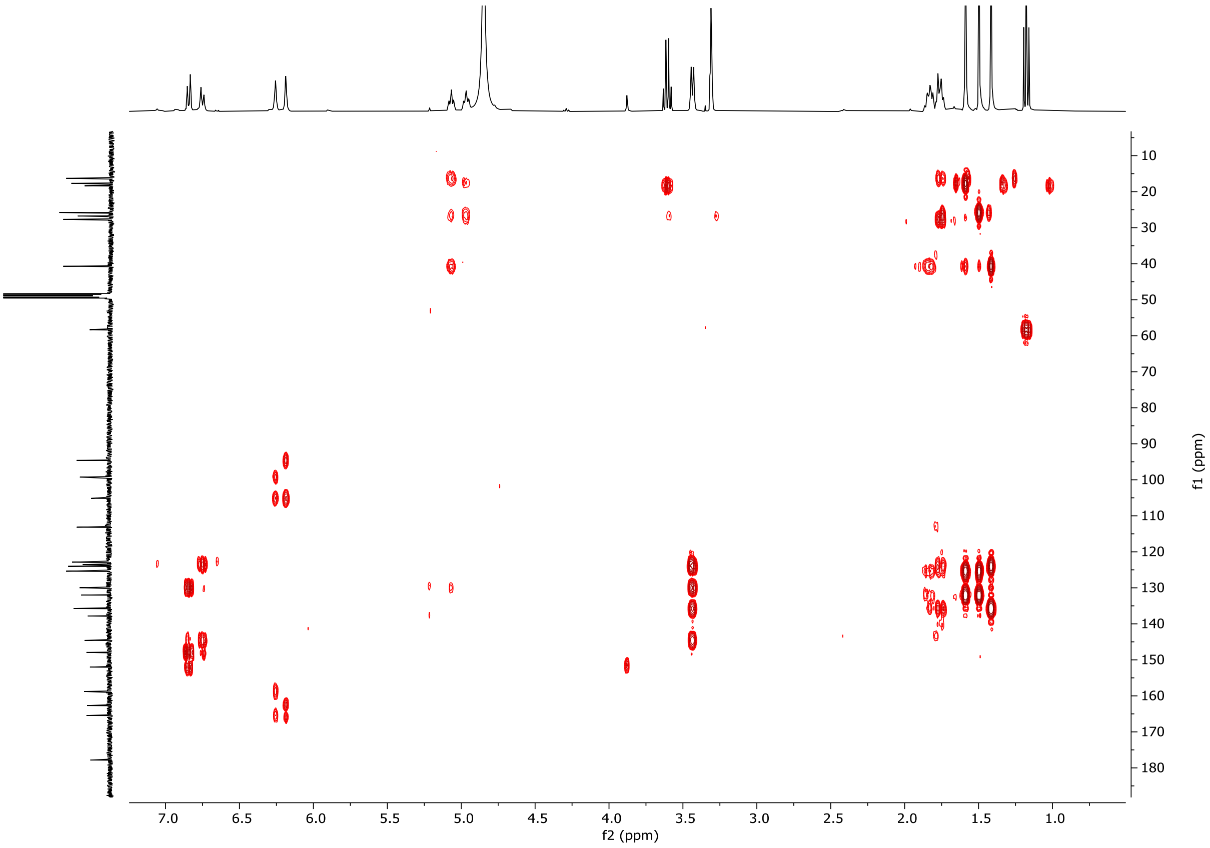

Figure S31. Two-dimensional diagrams showing non-covalent interactions of **1-9** and acarbose (**10**) with α-glucosidase (2ZQ0).

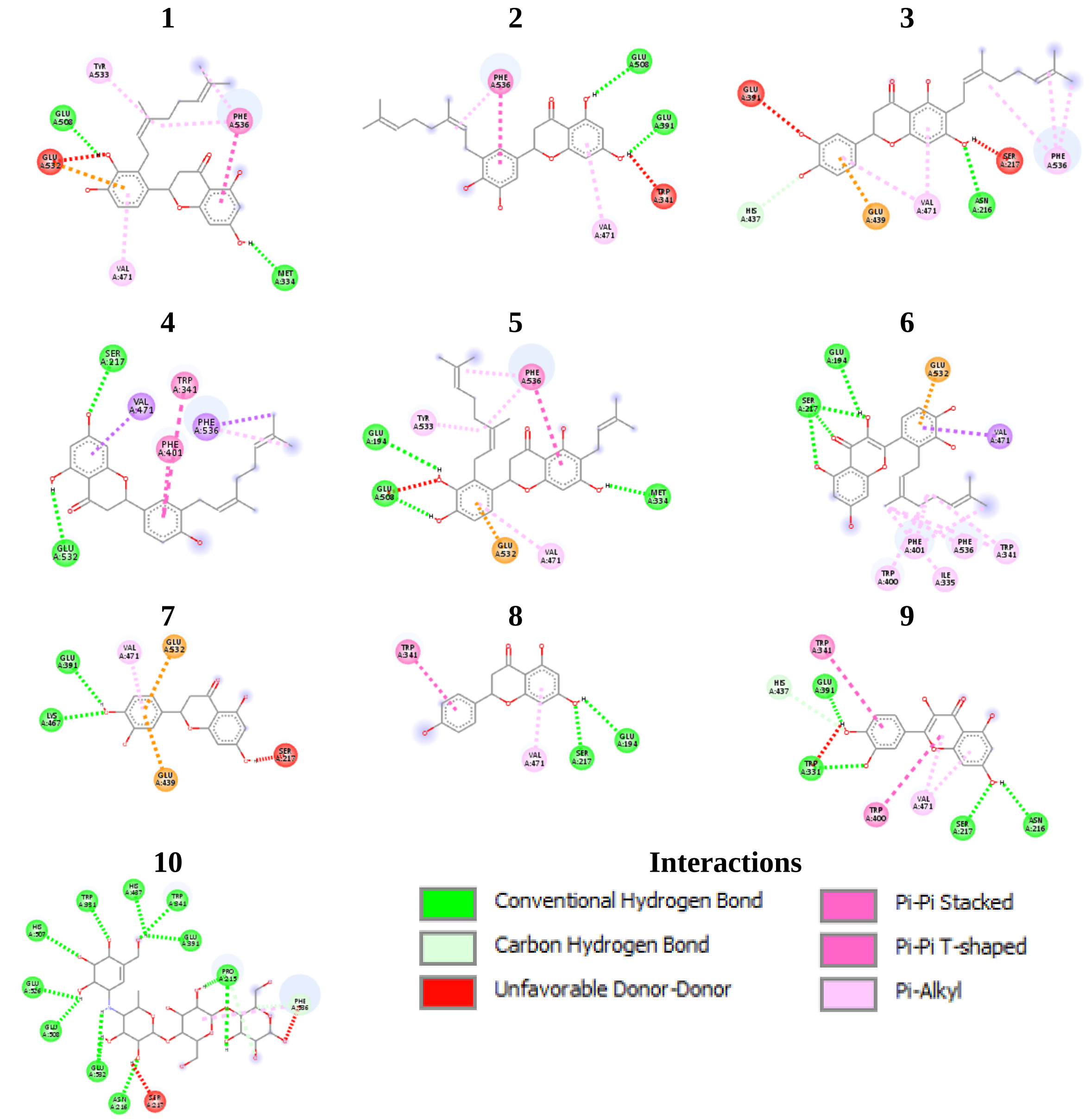

Figure S32. Two-dimensional diagrams showing non-covalent interactions of **1-9** and acarbose (**10**) with α-amylase (2QV4).

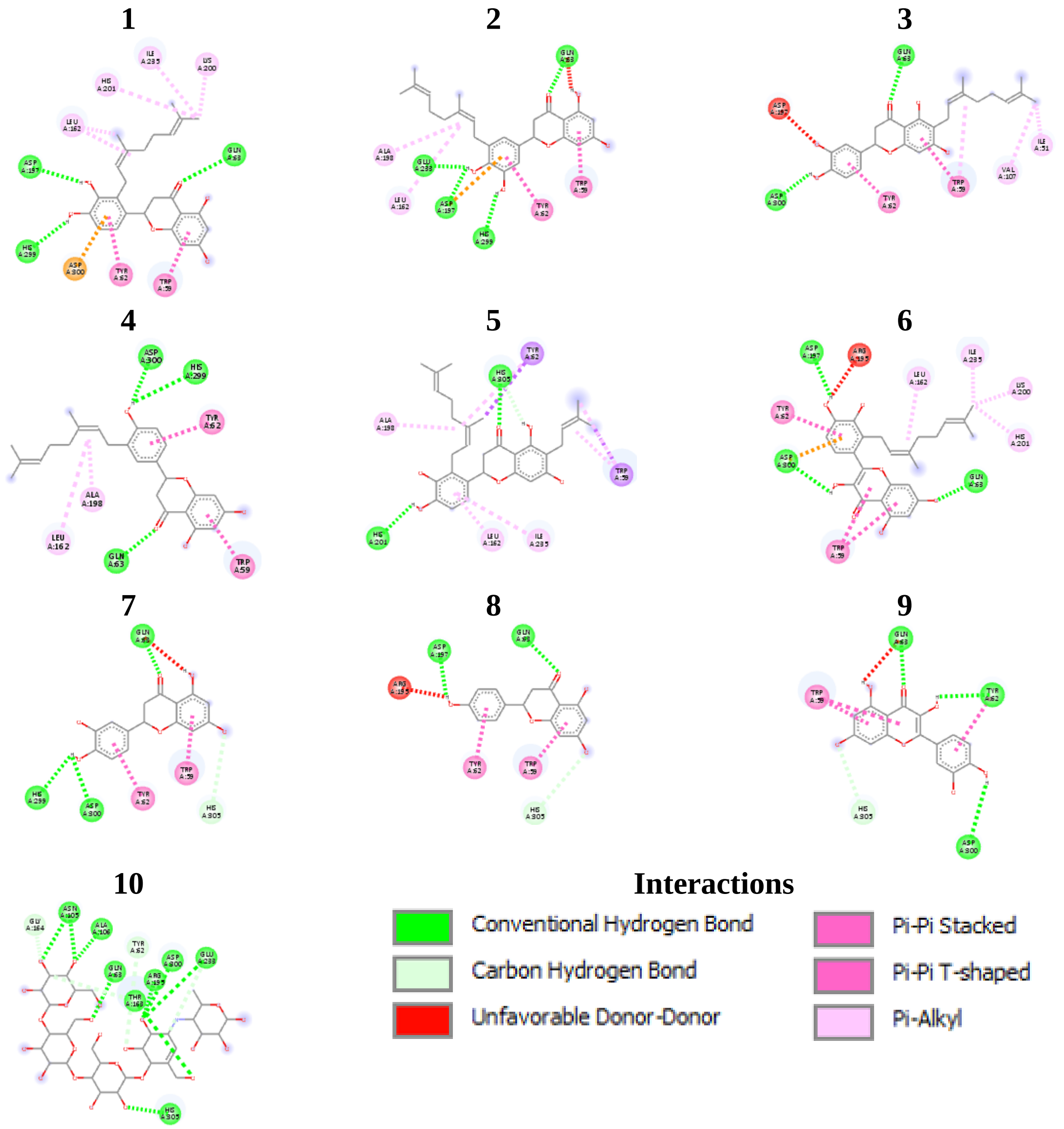

Figure S33. Two-dimensional diagrams showing non-covalent interactions of **1-9** and orlistat (**11**) with lipase (6KSM).

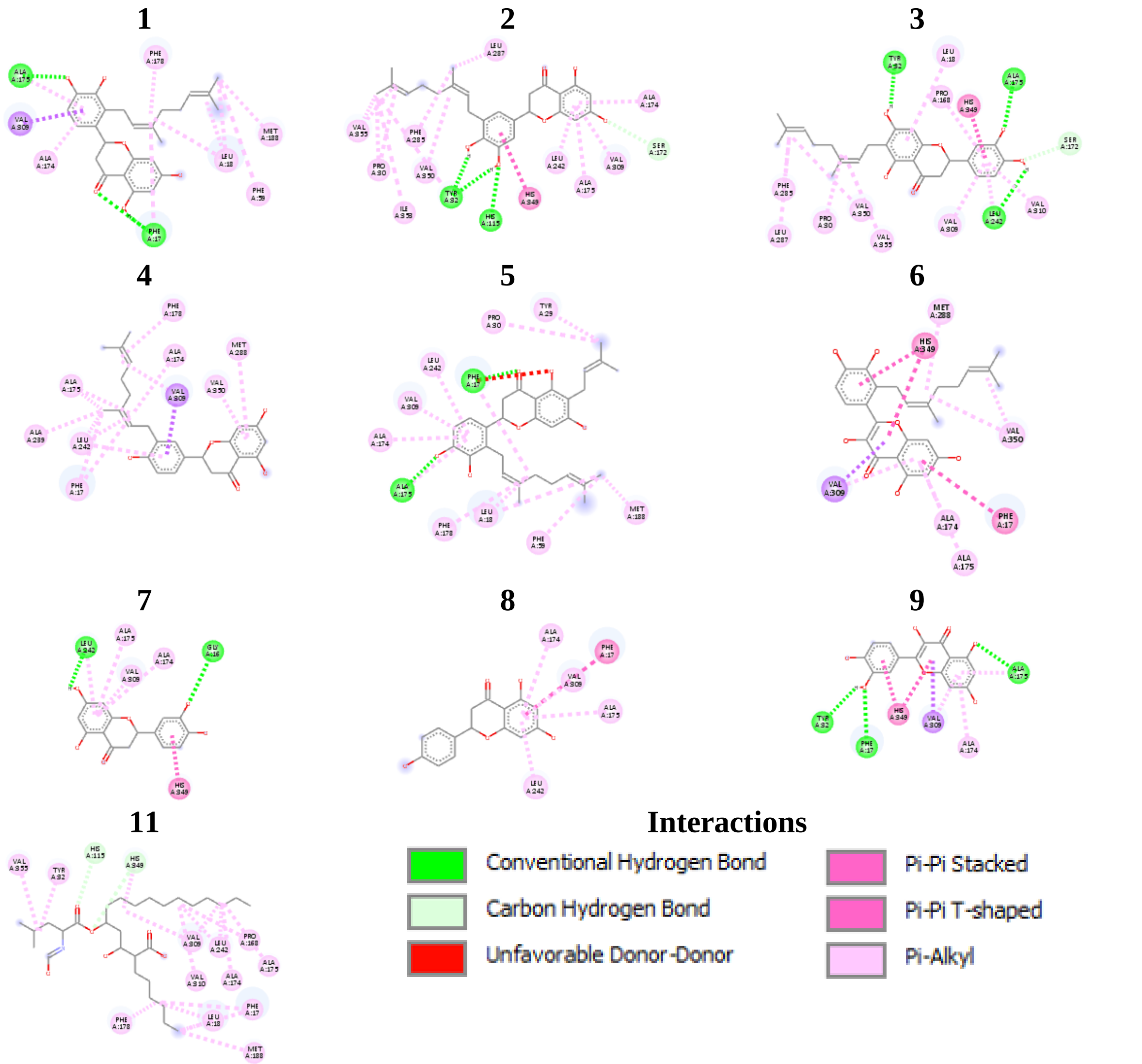

Supplement: Supplementary file 1 [file foods-11-01964-s001.zip › foods-1781639-Supplementary.pdf]
